# Integrated Analysis of Proteome and Transcriptome Profiling Reveals Pan-Cancer-Associated Pathways and Molecular Biomarkers

**DOI:** 10.1016/j.mcpro.2025.100919

**Published:** 2025-01-28

**Authors:** Guo-sheng Hu, Zao-zao Zheng, Yao-hui He, Du-chuang Wang, Rui-chao Nie, Wen Liu

**Affiliations:** 1 Biomedical Research Center of South China, College of Life Sciences, Fujian Normal University, Fuzhou, China; 2 State Key Laboratory of Cellular Stress Biology, School of Pharmaceutical Sciences, Faculty of Medicine and Life Sciences, Xiamen University, Xiamen, Fujian, China; 3 Fujian Provincial Key Laboratory of Innovative Drug Target Research, School of Pharmaceutical Sciences, Faculty of Medicine and Life Sciences, Xiamen University, Xiamen, Fujian, China; 4 Xiang An Biomedicine Laboratory, School of Pharmaceutical Sciences, Faculty of Medicine and Life Sciences, Xiamen University, Xiamen, Fujian, China; 5 MOE Key Lab of Rare Pediatric Diseases, Hengyang Medical School, University of South China, Hengyang, Hunan, China; 6 National Institute for Data Science in Health and Medicine, Xiamen University, Xiamen, Fujian, China

**Keywords:** pan-cancer, proteomics, transcriptomics, diagnostic marker, prognostic marker, drug targets

## Abstract

Understanding dysregulated genes and pathways in cancer is critical for precision oncology. Integrating mass spectrometry–based proteomic data with transcriptomic data presents unique opportunities for systematic analyses of dysregulated genes and pathways in pan-cancer. Here, we compiled a comprehensive set of datasets, encompassing proteomic data from 2404 samples and transcriptomic data from 7752 samples across 13 cancer types. Comparisons between normal or adjacent normal tissues and tumor tissues identified several dysregulated pathways including mRNA splicing, interferon pathway, fatty acid metabolism, and complement coagulation cascade in pan-cancer. Additionally, pan-cancer upregulated and downregulated genes (PCUGs and PCDGs) were also identified. Notably, RRM2 and ADH1B, two genes which belong to PCUGs and PCDGs, respectively, were identified as robust pan-cancer diagnostic biomarkers. TNM stage-based comparisons revealed dysregulated genes and biological pathways involved in cancer progression, among which the dysregulation of complement coagulation cascade and epithelial-mesenchymal transition are frequent in multiple types of cancers. A group of pan-cancer continuously upregulated and downregulated proteins in different tumor stages (PCCUPs and PCCDPs) were identified. We further constructed prognostic risk stratification models for corresponding cancer types based on dysregulated genes, which effectively predict the prognosis for patients with these cancers. Drug prediction based on PCUGs and PCDGs as well as PCCUPs and PCCDPs revealed that small molecule inhibitors targeting CDK, HDAC, MEK, JAK, PI3K, and others might be effective treatments for pan-cancer, thereby supporting drug repurposing. We also developed web tools for cancer diagnosis, pathologic stage assessment, and risk evaluation. Overall, this study highlights the power of combining proteomic and transcriptomic data to identify valuable diagnostic and prognostic markers as well as drug targets and treatments for cancer.

## Highlights

• Dysregulated genes and pathways were identified in cancer or across tumor stages.
• Pan-cancer up- and downregulated genes (PCUG, PCDG) were identified.
• RRM2 and ADH1B were emerged as robust pan-cancer diagnostic biomarkers.
• Pan-cancer continuously up- or downregulated proteins (PCCUP, PCCDP) were detected.
• Cancer diagnostic, pathologic, and prognostic models were developed as web tools.

## Graphical Abstract

**Figure:**
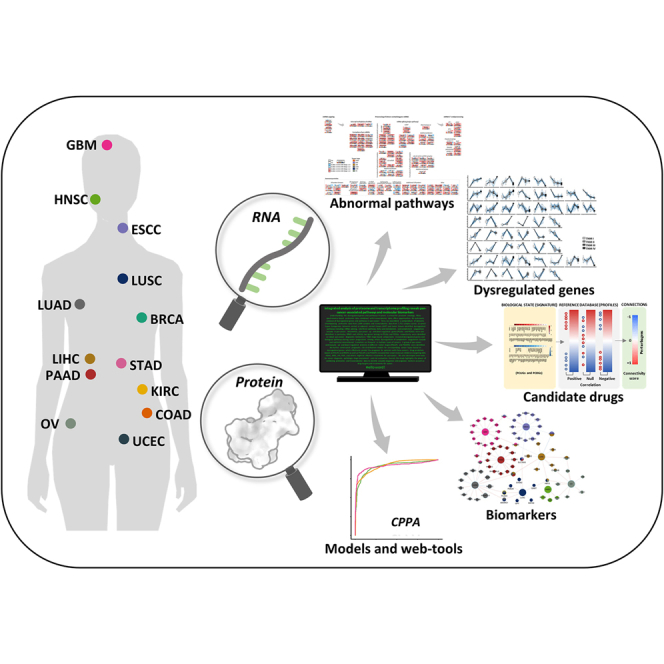

## In Brief

Proteomic and transcriptomic data across 13 cancer types were integrated to identify dysregulated pathways, such as splicing and interferon response. Pan-cancer dysregulated genes and proteins were identified to develop diagnostic, pathologic, and prognostic models, with RRM2 and ADH1B emerging as robust diagnostic biomarkers. Drug prediction suggested inhibitors targeting CDK, HDAC, and others may offer effective treatments for pan-cancer. This study highlights the power of combining proteome and transcriptome to identify biomarkers as well as drug targets for cancer.

Gene transcription is tightly regulated in normal cells. However, such regulation is disrupted in cancer cells, leading to aberrations in gene expression, including the overexpression of oncogenes and under-expression of tumor suppressor genes (1). The Cancer Genome Atlas (TCGA) project (2), launched in 2005, represents one of the most comprehensive multi-omics studies of cancer, covering thousands of samples from 33 cancer types (3, 4, 5, 6, 7, 8, 9, 10, 11, 12, 13, 14, 15, 16, 17). This project primarily focuses on genomic, epigenomic, and transcriptomic data, with the aim of enhancing our understanding of the molecular mechanisms underlying cancer development through multi-omics integration to uncover potential therapeutic targets. Indeed, numerous drug targets and/or pathways identified through this approach have proven effective in cancer treatment. However, there are often instances where treatments fail to elicit responses (18). One of the reasons is that genetic mutations and transcriptomic alterations do not always result in the predicted change of the corresponding protein, because they reside many regulatory layers away from the protein and there are many other factors that contribute to tumor behavior, such as protein modifications, metabolism, and the microbiome (18, 19).

Recent advancements in mass spectrometry (MS) technology have paved the way for large-scale investigation of the cancer proteome. The Clinical Proteomic Tumor Analysis Consortium (CPTAC) (20) is a pivotal project dedicated to accelerate the understanding of the molecular basis of cancer through large-scale multi-omics data, with proteomics serving as the core modality. To date, CPTAC has covered over 2000 patients from 15 different types of cancer so far (21, 22, 23, 24, 25, 26, 27, 28, 29, 30, 31). Additionally, recent studies have highlighted the molecular characteristics and potential therapeutic targets of cancers by combining proteomics with genomics (32, 33, 34).

Pan-cancer analysis, which involves comprehensive research on multiple cancer types, aims to identify commonalities and specificities among various cancers to more thoroughly understand cancer occurrence, progression, and treatment. Recently, the CPTAC has published summary research projects: the pan-cancer atlas. For instance, pan-cancer analysis based on multi-omics has identified cis-effects and distal trans-effects at RNA, protein, and phosphoprotein, revealing the impacts on oncogenic drivers (35). Tthe exploration of posttranslational modifications of pan-cancer has unveiled the shared and unique regulatory patterns of posttranslational modifications, potentially paving the way for new therapeutic avenues (36). Proteogenomic analysis of pan-cancer has illustrated the immune landscape, identifying seven distinct immune subtypes and the corresponding molecular characteristics to develop future immunotherapy and precision medicine strategies (37). The standardized datasets (https://pdc.cancer.gov/pdc/) (38) and web tools (LinkedOmics and LinkedOmicsKB) (39, 40) have been established based on multi-omics data of pan-cancer to promote data reuse. In addition, several research institutions and laboratories have published significant findings centered on pan-cancer proteomics. For example, pan-cancer analysis based on proteomics and transcriptomics has underscoring the importance of a comprehensive understanding of the posttranscriptional regulatory landscape of cancer (41). Pan-cancer has been divided into 11 subtypes based on proteomics to facilitate the development of personalized treatment strategies (42). The pan-cancer associated tumor-enriched and highly expressed cell surface antigens have been identified as potential targets through proteomics for the development of innovative therapeutics (43).

RNAs and proteins are not only crucial functional players but also closely interconnected in abundance within cells. Numerous studies have demonstrated that the integrated analysis of proteomics and transcriptomics not only enables more accurate and effective identification and validation of disease biomarkers (44, 45) but also allows for a more comprehensive and systematic exploration of the biological perturbations during disease development (46, 47). Furthermore, it facilitates the prediction of patient survival outcomes, treatment responses, and drug resistance, thus promoting the development of personalized treatment strategies (48, 49). Therefore, the integrated analysis of pan-cancer transcriptomic and proteomic data can enhance the complementation and integration of mRNA and protein, enabling a more accurate and holistic understanding of the perturbations in biological pathways and biomarkers in cancer from another perspective and offering a clearer perspective on cancer treatment.

In this work, we comprehensively analyzed transcriptomic and proteomic data from 10,156 samples, including normal tissues, adjacent normal tissues (ANTs), and primary tumors across 13 distinct cancer types. We identified common dysregulated genes and pathways among different cancer types and during cancer development. Furthermore, we identified diagnostic markers, prognostic markers, and potential treatments for cancer. Finally, we constructed web tools for cancer diagnosis, pathologic stage classification, and prognostic risk stratification.

## Experimental Procedures

### Proteomic Datasets

A compendium dataset of MS-based proteomic data included 13 different types of cancers and covering over 2000 samples and nearly 1500 patients (Supplemental Table S1). The cancer types included in the proteomic dataset were as following: breast cancer (cancer samples, n = 124; non-cancer samples, n = 18), colorectal cancer (cancer samples, n = 97; non-cancer samples, n = 100), esophageal squamous cell carcinoma (cancer samples, n = 124; non-cancer samples, n = 124), glioblastoma (cancer samples, n = 99; non-cancer samples, n = 10), head and neck squamous cell carcinoma (cancer samples, n = 108; non-cancer samples, n = 67), clear cell renal cell carcinoma (cancer samples, n = 110; non-cancer samples, n = 84), hepatocellular carcinoma (cancer samples, n = 165; non-cancer samples, n = 165), lung adenocarcinoma (cancer samples, n = 110; non-cancer samples, n = 101), lung squamous cell carcinoma (cancer samples, n = 108; non-cancer samples, n = 100), ovarian cancer (cancer samples, n = 83; non-cancer samples, n = 20), pancreatic ductal adenocarcinoma (cancer samples, n = 140; non-cancer samples, n = 67), gastric cancer (cancer samples, n = 80; non-cancer samples, n = 80), and endometrial carcinoma (cancer samples, n = 95; non-cancer samples, n = 25).

All proteomic data were downloaded from the CPTAC data portal or PRIDE (https://www.ebi.ac.uk/pride/) and have been recomputed through a standard pipeline to minimize the abiologic difference. MaxQuant software (version 2.0.2.0) was used to analyze MS raw files (50). MS/MS spectra were searched against the reviewed SwissProt human proteome database containing 20,386 proteins (downloaded on August 29, 2021) and a common contaminants database by the Andromeda search engine (51). If there is a “internal reference,” such as a mixed sample, the reference channel will be set according to the corresponding plex, and the normalization method will be set as “Weighted ratio to reference channel.” Carbamidomethylation was applied as fixed and N-terminal acetylation, deamidation at NQ, and methionine oxidation as variable modifications. Enzyme specificity was set to “Trypsin/P” or “Trypsin/P + LysC” with a maximum of two missed cleavages and a minimum peptide length of seven amino acids according to corresponding published papers. An false discovery rate (FDR) of 1% was applied at the peptide and protein level. Peptide identification was performed with an allowed initial precursor mass deviation of up to 7 ppm and an allowed fragment mass deviation of 20 ppm. Protein identification required at least 1 “razor + unique peptides.” Data were filtered for common contaminants, and peptides only identified by side modification were excluded from further analysis. To mitigate systematic and sample-specific bias in the quantification, the expression ratios were log2-transformed and normalized using the median-centering method across proteins. The protein quantification data can be downloaded from CPPA web tools *via* the following link: (https://www.cppa.site/sysproteome/sysproteome_download) or iProX database (accession number IPX0010644001) (52). Clinical information was downloaded from the CPTAC data portal or obtained from published papers. Major clinical parameters, including clinical stage, pathological stage, histological grade, age, race, gender, survival time, and vital status of all cancer patients, were further organized into structured data tables.

### Transcriptomic Datasets

For transcriptomic datasets sourced from CPTAC, which are corresponding to proteomic data, gene expression data (read counts data and TPM normalized data) and clinical information were directly downloaded from the Genome Data Commons, containing 10 cancer types and covering 1502 samples (Supplemental Table S1). The cancer types included in the transcriptomic datasets sourced from CPTAC were as following: breast cancer (cancer samples, n = 120), colorectal cancer (cancer samples, n = 106), glioblastoma (cancer samples, n = 99; non-cancer samples, n = 9), head and neck squamous cell carcinoma (cancer samples, n = 110; non-cancer samples, n = 61), clear cell renal cell carcinoma (cancer samples, n = 110; non-cancer samples, n = 75), lung adenocarcinoma (cancer samples, n = 111; non-cancer samples, n = 102), lung squamous cell carcinoma (cancer samples, n = 108; non-cancer samples, n = 95), ovarian cancer (cancer samples, n = 101), pancreatic ductal adenocarcinoma (cancer samples, n = 140; non-cancer samples, n = 39), and endometrial carcinoma (cancer samples, n = 101; non-cancer samples, n = 15).

For transcriptomic datasets sourced from TCGA-GTEx, gene expression data (read counts data and TPM normalized data) were downloaded from the UCSC-XENA (53, 54), including 13 cancer types and covering 6241 samples (Supplemental Table S1). The clinical information of samples from TCGA were downloaded from Genome Data Commons. The cancer types included in the transcriptomic datasets sourced from TCGA-GTEx were as following: breast cancer (cancer samples, n = 1099; non-cancer samples, n = 113), colorectal cancer (cancer samples, n = 289; non-cancer samples, n = 41), esophageal squamous cell carcinoma (cancer samples, n = 92; non-cancer samples, n = 13), glioblastoma (cancer samples, n = 166; non-cancer samples, n = 206), head and neck squamous cell carcinoma (cancer samples, n = 520; non-cancer samples, n = 44), clear cell renal cell carcinoma (cancer samples, n = 530; non-cancer samples, n = 72), hepatocellular carcinoma (cancer samples, n = 371; non-cancer samples, n = 50), lung adenocarcinoma (cancer samples, n = 515; non-cancer samples, n = 59), lung squamous cell carcinoma (cancer samples, n = 498; non-cancer samples, n = 50), ovarian cancer (cancer samples, n = 426; non-cancer samples, n = 88), pancreatic ductal adenocarcinoma (cancer samples, n = 179; non-cancer samples, n = 167), gastric cancer (cancer samples, n = 413; non-cancer samples, n = 36), and endometrial carcinoma (cancer samples, n = 181; non-cancer samples, n = 23).

### Tissue-Specific Genes Analysis

Tissue-specific genes in the Human Protein Atlas (HPA) database are defined as genes that are highly expressed in a particular tissue or organ, with significantly higher expression levels in that tissue than others. The HPA classifies tissue-specific genes using large-scale RNA-Seq data combined with antibody-based protein detection through immunohistochemistry. Based on gene expression levels in different tissues, the HPA categorizes tissue-specific genes into the following classes: tissue enriched (at least four-fold higher mRNA level in a particular tissue than any other tissue), group enriched (at least four-fold higher average mRNA level in a group of 2–5 tissues than any other tissue), and tissue enhanced (at least four-fold higher mRNA level in a particular tissue than the average level in all other tissues). In this study, the relevant tissue-specific genes were downloaded from the HPA (https://www.proteinatlas.org/humanproteome/tissue/tissue+specific) and selected the most representative "Tissue enriched" category to explore the expression differences of tissue-specific genes between normal and tumor tissues.

### Differential Expression Analysis

For comparison of protein expression between cancer and non-cancer samples, two-sided wilcoxon rank-sum test was performed to assess the statistical significance. Proteins with *Q* value (Benjamini-Hochberg (BH) adjusted *p* value) < 0.01 and fold change (FC, the ratio of median abundance of proteins between cancer and non-cancer samples) > 1.2 and <0.83 were considered to be significantly upregulated and downregulated, respectively, in cancer samples. Similarly, the R package edgeR (55) was applied to calculate the FDR of differentially expressed mRNA for transcriptomic data. mRNAs with *Q* value (FDR) < 0.01 and FC > 1.5 and < 0.67 were considered to be significantly upregulated and downregulated in cancer samples.

For comparison of protein expression between TNM stages, ANOVA was employed to assess the statistical significance. *p* value < 0.05 were considered to be significantly changed proteins during the progression of cancer.

### T-SNE Analysis

t-distributed stochastic neighbor embedding (t-SNE) is a dimensionality reduction technique and data visualization method. The t-SNE analysis performed by Rtsne (version 0.16) was used for quality check of proteomic and transcriptomic data.

### Functional Enrichment Analysis

Functional enrichment analysis was performed using R package clusterProfiler (version 4.6.2) (56) to identify KEGG pathways, wikiPathways, Hallmark gene sets, and gene ontology biological functions that enriched for different gene or protein sets. The following categories are included: (1) upregulated genes at both RNA and protein level in each cancer type; (2) downregulated genes at both RNA and protein level in each cancer type; (3) pan-cancer upregulated genes (PCUGs); (4) pan-cancer downregulated genes (PCDGs); (5) proteins in the trend clusters from each cancer type; (6) pan-cancer continuously upregulated proteins (PCCUPs); (7) pan-cancer continuously downregulated proteins (PCCDPs). The statistical significance (*p* value) was evaluated by hypergeometric test.

### Protein-Protein Interaction Enrichment Analysis

Protein-protein interaction enrichment analysis for glioblastoma (GBM) tissue was performed by Metascape (57). The network diagram contains the subset of proteins that form physical interactions with at least one other member in the list. The Molecular Complex Detection algorithm has been applied to identify densely connected network components. Pathway and process enrichment analysis has been applied to each Molecular Complex Detection component independently. Protein–protein interaction analysis for the network of PCUGs and PCDGs, as well as PCCUPs and PCCDPs, was performed by STRING (58) and the visualization of networks was performed by Cytoscape (59).

### Correlation of mRNA-Protein and Gene Set Enrichment Analysis

Gene set enrichment analysis (GSEA) is a computational method that determines whether an *a priori* defined set of genes shows statistically significant, concordant differences between two biological states. Gene-wise Spearman correlation of protein and mRNA was performed on 9057 genes across 1429 samples, which was used as the input of GSEA analysis. GSEA performed by clusterProfiler (56) was used for pathway enrichment analysis of gene-wise spearman correlation based on biological process aspect from gene ontology. The following parameters were used to run GSEA; by: fgsea; nPerm: 1000; minGSSize: 50; maxGSSize: 1000; pAdjustMethod: BH; pvalueCutoff: 0.05.

### CMap-Based Drug Prediction

The connectivity Map (CMap) is a resource that uses transcriptional expression data from cultured human cells treated with perturbagens to probe relationships between diseases, cell physiology, and therapeutics. Each reference gene-expression profile in CMap is represented as a rank-ordered gene list. The query signature is compared to each rank-ordered list to determine whether upregulated proteins tend to appear near the top of the list and downregulated proteins near the bottom, which is defined as “positive connectivity” and otherwise “negative connectivity.” We first constructed query signatures and then mapped the query signatures to CMap (60, 61). To predict candidate drugs for cancer patients, gene sets, including PCUGs and PCDGs as well as PCCUPs and PCCDPs, were selected as the query signatures. The connectivity score of each perturbagen was calculated using the query signature. We sorted perturbagens according to their connectivity scores in increasing order. As a high negative connectivity score indicates that the corresponding perturbagen reversed the expression of the query signature, the top drugs with the highest negative connectivity scores were predicted as potential drugs.

### Single-Sample Gene Set Enrichment Analysis

Single-sample gene set enrichment analysis (ssGSEA) was performed using R package GSVA (version 1.46.0) (62) to calculate the ssGSEA scores for complement and coagulation gene set and epithelial-mesenchymal transition (EMT) gene set. The gene sets used in the ssGSEA analysis were downloaded from the Molecular Signatures Database database.

### Expression Trend Analysis

Fuzzy clustering of time-series expression data is a highly useful technique for analyzing temporal data. We used R package Mfuzz (version 2.58.0) (63) to cluster temporal gene expression data during the progression of cancer. When cancer samples contain TNM stage IV, the number of trend clusters is specified as 8. Otherwise, the number of clusters is specified as 6. The following parameters were used to run Mfuzz; c: 6 or 8, m: calculated by *mestimate* (function contained in Mfuzz), iter.max: 1000.

### Pan-Cancer Diagnostic Model

#### Diagnostic Models for Pan-Cancer

The abundance of RRM2 and ADH1B were separately normalized by z-score transformation in both transcriptomic and proteomic data. Ten machine learning algorithms were respectively applied with 100 rounds of five-fold cross-validation (CV) to calculate receiver operating characteristics (ROC)-area under the curve (AUCs) by R package mlr3verse (version 0.2.8) for proteomic data or transcriptomic data soured from TCGA-GTEx for building diagnostic model. ROC-AUC value was used as the criterion to pick the best machine learning algorithm and diagnostic model. The transcriptomic data from differential source was used as testing cohort to validate diagnostic model built by proteomic data, and transcriptomic and proteomic data sourced from CPTAC were used for validating diagnostic model built by transcriptomic data sourced from TCGA-GTEx. The following machine learning algorithms were used to build models: Logistic Regression (Log.Reg), Linear Discriminant Analysis (LDA), Naïve Bayes (Naïve.Bayes), k-nearest neighbor (KNN), Support Vector Machine (SVM), Neural Network (Neural.Net), light Gradient Boosting Machine (lightGBM), Gradient Boosting Decision Tree (XGBoost), Recursive Partitioning and Regression Trees (RPaRT), Random forest.

#### Diagnostic Models for Echo Tumor Type

The same process as described above was used, and the analysis was done for each cancer type.

### Cancer Stage Classification Model

#### Feature Selection

To identify genes that classify patients in early and advanced stages, the differential expressed genes in early *versus* advanced stage were used as the initial feature set for candidate feature identification. Feature selection was implemented on the initial feature set using the mlr3verse. In order to facilitate clinical utility, we limit the maximum number of features to no more than 5. SVM algorithms were used as the classifier because it is characterized by fair generalization ability. Other parameters were set as follows: feature selection method = “sfs” (sequential forward search), resampling algorithm = “Subsample,” number of resampling = 30, and performance measures = “auc.” We set the maximum number of features (“max.features”) to 1, 2, 3, 4, or 5 and repeated the feature selection process 100 times. Feature sets with top five frequency in each maximum feature number were selected as the candidate feature sets.

#### Model Selection

According to candidate feature sets, we applied 10 machine learning algorithms with 100 rounds of five-fold CV to calculate ROC-AUCs by mlr3verse. The transcriptomic data sourced from CPTAC was used as testing cohort to validate stage classification models. ROC-AUC value was used as the criterion to select top 10 models and feature sets. Finally, taking the number of features, stability of model, and prediction performance into consideration, best stage classification biomarkers and models for each cancer type was selected.

### Cancer Prognostic Risk Stratification Model

#### LASSO-Cox Regression Model

Proteomic data was used as training cohort, and transcriptomic data from different source was regarded as testing cohort. Based on the PCUGs, PCDGs, PCCUPs, and PCCDPs gene sets, we employed Least Absolute Shrinkage and Selector Operation (LASSO)-Cox regression analysis to identify the prognostic risk gene set for each cancer type in proteomic data by R package glmnet (version 4.1–6) (64). The following parameters were used to run LASSO-Cox. Family: "cox," alpha: 1, nlambda: 1000, standardize: TRUE. The risk score for every cancer patient was calculated based on the following formula:

$$\text{Risk}\;\text{score}=\sum_{i=0}^{n}G_{i}F_{i}$$

Where *n* was the number of genes in the risk gene set, *Gi* was the normalized expression value of gene *i*, and *Fi* was the regression coefficient of gene *i* in the LASSO-Cox regression analysis. As a cut-off value, the median risk score divided cancer samples into the low- and high-risk groups. Likewise, we investigated the risk score in predicting patients’ survival on transcriptomic data.

#### Model Selection

According to risk gene sets in each cancer type, we applied 10 machine learning algorithms with 100 rounds of five-fold CV to calculate ROC-AUCs by mlr3verse. The transcriptomic data was used as testing cohort for validation. ROC-AUC value was used as the criterion to select the best model.

### Time-dependent ROC Curve

As clinical outcomes are time-dependent, we used time-dependent ROC curve for censored data and ROC-AUC implemented in the R package survivalROC (version 1.0.3.1) to evaluate the predictive performance of prognostic risk stratification models. Larger ROC-AUCs at time *t* indicates better predictability of time to event (patient risk) at time *t*. We plotted ROC-AUCs ranging from 1/4 to 5 years to compare the overall predictive performance of prognostic models.

### Data Visualization

Data visualization was performed in R (version 4.2.2), using the ggplot2 (version 3.4.1), ggpubr (version 0.6.0), ggraph (version 2.1.0), pheatmap (version 1.0.12), and UpSetR (version 1.4.0) packages.

## Results

### Compendium of Transcriptomic and Proteomic Datasets

**Fig. 1:**
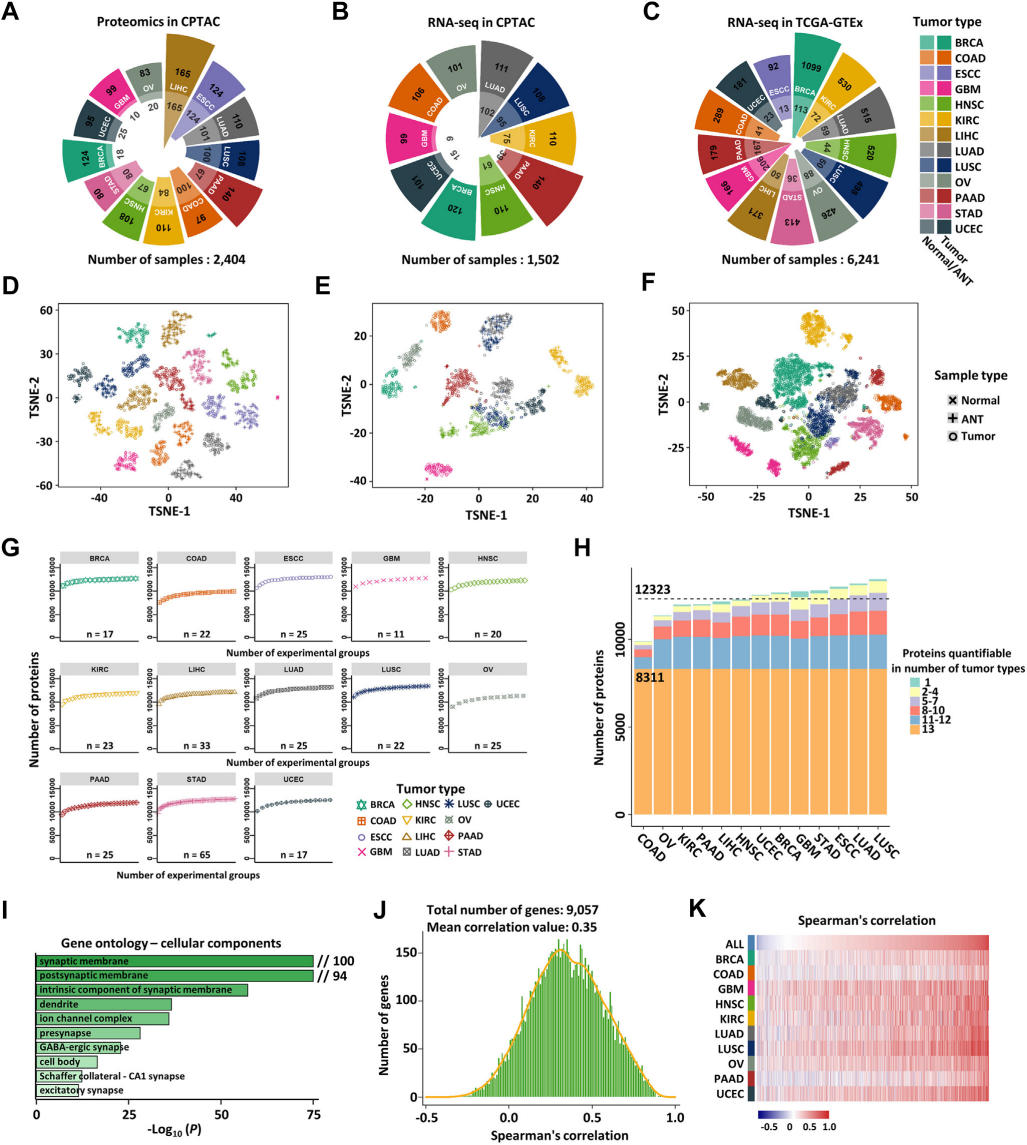
**Compendium of transcriptomic and proteomic datasets**. *A*–*C*, summary of proteomic data obtained from CPTAC (*A*) and transcriptomic data obtained from CPTAC (*B*) and TCGA-GTEx (*C*). Cancer types are represented by distinct colors. Within each cancer type, normal or adjacent normal tissues (ANTs) are shown by lighter shades, while tumor tissues by darker shades. *D*–*F*, t-SNE analysis of proteomic data obtained from CPTAC (*D*) and transcriptomic data obtained from CPTAC (E) and TCGA-GTEx (*F*). The cross, plus, and circle represent normal, ANTs, and tumor tissues, respectively. *G*, cumulative number of proteins quantified in each cancer type. *H*, distribution of the number of cancer types in which the proteins were quantifiable. An average of 12,323 proteins were quantified, and 8311 proteins were quantified in all cancer types. *I*, gene ontology (GO) analysis of cellular component for unique proteins in GBM tissues. *J*, histogram of gene-wise (n = 9057) Spearman correlation (mean = 0.35) between mRNA and protein levels in 1429 samples. *K*, heatmap of gene-wise Spearman correlations of mRNA and protein levels in each cancer type.

We assembled a comprehensive dataset of transcriptomic data and MS-based proteomic data, covering 13 types of tumor tissues and corresponding normal tissues or ANTs from patients with breast cancer (BRCA), colorectal cancer (COAD), esophageal squamous cell carcinoma (ESCC), GBM, head and neck squamous cell carcinoma (HNSC), clear cell renal cell carcinoma (KIRC), hepatocellular carcinoma (LIHC), lung adenocarcinoma (LUAD), lung squamous cell carcinoma (LUSC), ovarian cancer (OV), pancreatic ductal adenocarcinoma (PAAD), gastric cancer (STAD), and endometrial cancer (UCEC). This dataset includes proteomic data from 2404 samples and corresponding transcriptomic data from 1502 samples sourced from the CPTAC project, as well as transcriptomic data from 6241 samples sourced from the TCGA and GTEx projects (Fig. 1, *A*–*C* and Supplemental Table S1). For the transcriptomic and proteomic datasets, we normalized expression values within each cancer type to ensure that tissue-specific differences and interlaboratory batch effects will not affect downstream analyses. These three datasets effectively differentiate at tissue level as well as tumor and non-tumor level through t-SNE analysis (Fig. 1, *D*–*F*). For the proteomic data, a total of 16,623 proteins were identified, of which 9059 proteins were quantifiable in more than half of the samples, and 3630 proteins were quantifiable across all samples in all cancer types (Supplemental Fig. S1*A*). These proteins are primarily involved in fundamental cellular processes such as cytoplasmic translation, RNA splicing, protein folding, among others (Supplemental Fig. S1*B*). The cumulative number of proteins within each cancer type approached a plateau with an increasing number of experimental groups, suggesting that samples included adequately captured the proteomic landscape of the respective cancer type (Fig. 1*G*). The average number of quantified proteins across the 13 cancer types was 12,323, with 8311 proteins being quantifiable in all cancer types (a protein is considered quantifiable for a specific cancer type if it has quantitative information in at least one sample from that type) (Fig. 1*H*). The largest number of unique proteins was detected in GBM (Fig. 1*H*), which are primarily concentrated in proteins functioning in physiological structures of the brain, such as synaptic/postsynaptic membranes, dendrite, and ion channel complex, among others (Fig. 1*I* and Supplemental Fig. S1*C*). We integrated the above-mentioned proteomic datasets into CPPA web tool, allowing users to query and explore the protein of interests (https://cppa.site/cppa) (65).

We next sought to explore the correlation between the transcriptomic and proteomic data. In general, protein levels are broadly correlated with the corresponding mRNA level. For 9057 genes from 1429 samples that are overlapped in both the transcriptomics and proteomics studies, the median Spearman correlation of gene-wise protein *versus* mRNA was 0.35 (Fig. 1, *J* and *K*), with 8097 genes having significant positive correlations (Spearman correlation *p* < 0.01). The genes that showed strong correlation at protein and mRNA levels were mainly concentrated in biological processes such as cell-cell junction and amino acid catabolic process (Supplemental Fig. S1*E*), which is consistent with previous reports (41, 42). The correlation between protein and mRNA varied among different cancer types, with the best correlation observed in GBM, LUSC, UCEC, and HNSC and the worst in PAAD, BRCA, and COAD (Fig. 1*K* and Supplemental Fig. S1*F*).

### Dysregulated Genes and Pathways Were Identified by Transcriptomic and Proteomic Analysis

**Fig. 2:**
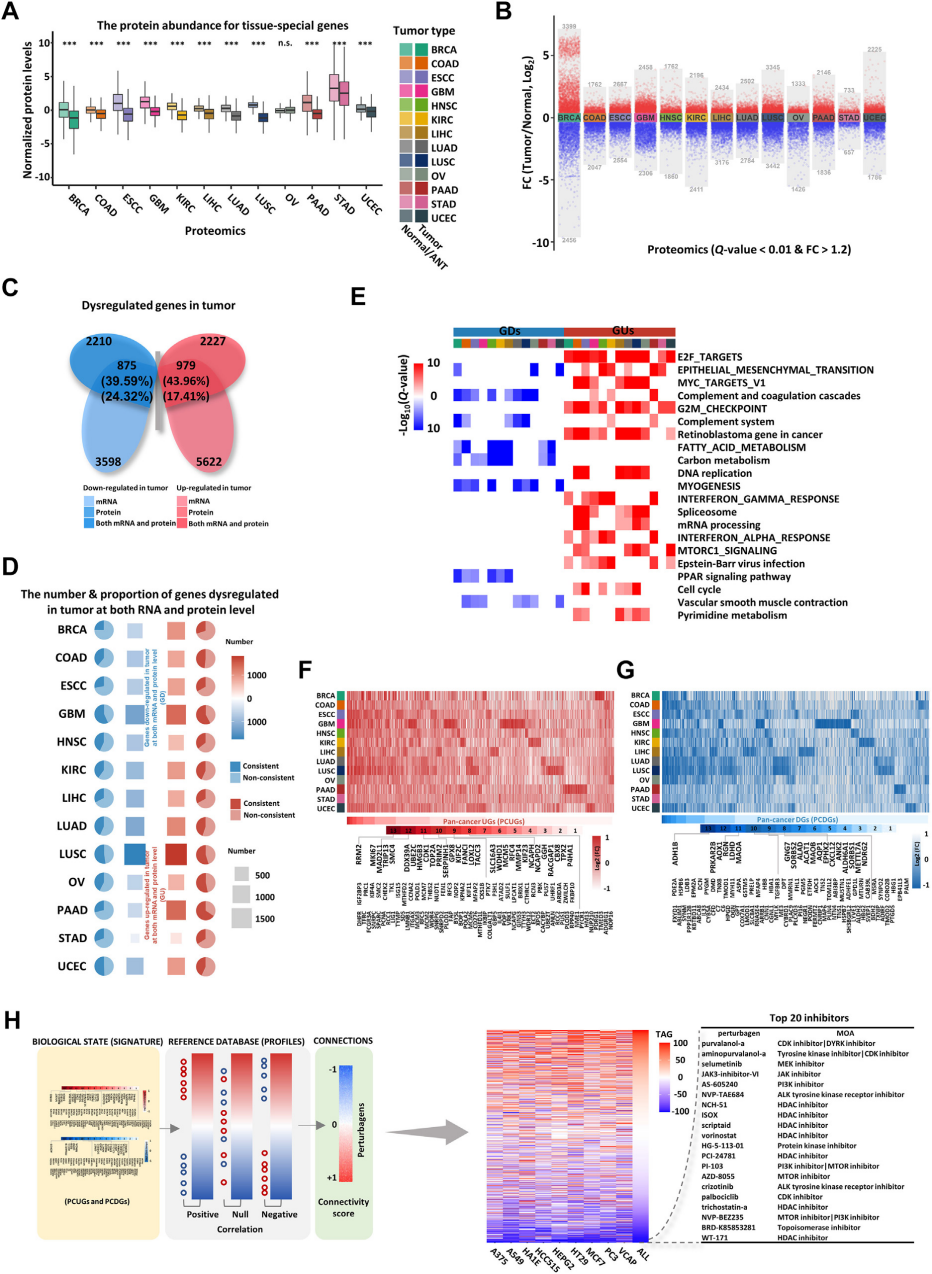
**Dysregulated genes and pathways were identified by transcriptomic and proteomic analysis**. *A*, the expression of tissue-specific proteins is shown by Boxplot. For each cancer type, normal or adjacent normal tissues (ANTs) are shown by lighter shades, while tumor tissues by darker shades. *p* values were calculated by two-sided wilcoxon rank-sum test. The middle bar represents the median, and the box represents the interquartile range. Bars extend to 1.5 × the interquartile range (∗*p* < 0.05, ∗∗*p* < 0.01, ∗∗∗*p* < 0.001, n.s.: non-significant). *B*, differential expression analysis showing upregulated and downregulated proteins across all 13 cancer types. *Red* and *blue* colors represent proteins that are upregulated and downregulated in tumor tissues, respectively (*Q* value (Benjamini-Hochberg (BH) adjusted *p* value) < 0.01 and fold change (FC) > 1.2). The number of differentially expressed proteins is indicated. *p* value was calculated by two-sided wilcoxon rank-sum test. *C*, the average number of upregulated and downregulated proteins and mRNAs is shown by Venn diagram. Genes that are upregulated and downregulated are represented by *red* and *blue* color, respectively. mRNAs and proteins are represented by lighter and darker shades, respectively. *D*, the number and proportion of genes that are upregulated and downregulated at both mRNA and protein levels (UGs and DGs) in each cancer type. UGs and DGs are represented by *red* and *blue* color, respectively. *E*, the significance of enrichment (by over representation analysis method in R package clusterProfiler) for gene sets from Hallmark, KEGG, and wikiPathway with UGs and DGs in each cancer type is shown by heatmap. The *Q* value of the gene set must be less than 0.001, the number of intersections with UGs and DGs must be at least 15, and the gene set must be enriched by more than five cancer types. The enrichment results of UGs and DGs are represented by *red* and *blue* color, respectively. *F* and *G*, the expression of genes that are upregulated and downregulated in tumor at both RNA and protein level (GUs and GDs) in each cancer type is shown by heatmap, and pan-cancer upregulated and downregulated genes (PCUGs and PCDGs) are shown at the *bottom*. *H*, the workflow of drug prediction based on PCUGs and PCDGs. The 117 PCUGs and 102 PCDGs were used as the query signature to match the reference profiles of perturbagens in CMap to calculate connectivity scores. Perturbagens are sorted by connectivity score in increasing order, and the *top* perturbagens are predicted as candidate drugs.

As expected, underexpression of tissue-specific genes at both mRNA and protein level was detected in all types of cancers (Fig. 2*A* and Supplemental Fig. S2*A*) (66). For example, GABBR2, a GABA receptor subunit, was specifically downregulated in GBM, and NPHS2, a molecule crucial for maintaining the structure and function of the glomerular filtration barrier, was underpresented in KIRC (Supplemental Fig. S2*B*). However, the overall protein abundance between normal and tumor was not changed across all cancer types (Supplemental Fig. S2*C*). These findings suggested that the loss of tissue identity is a characteristic of cancer. We next sought to identify genes that were differentially expressed between normal and tumor tissues. Considering the ratio compression effect in isobaric tag-based proteomic data (67), *Q* value (BH adjusted *p* value) < 0.01 and FC ≥ 1.2 were applied as thresholds to identify differentially expressed proteins. Similarly, *Q* value < 0.01 and FC ≥1.5 were used as thresholds to screen differentially expressed mRNAs. A substantial number of differentially expressed proteins and mRNAs were identified in cancers (Fig. 2*B* and Supplemental Fig. S2*D*). Furthermore, upregulated or downregulated genes in tumor at both protein and RNA levels were identified in each cancer type (UGs and DGs) (Fig. 2, *C* and *D*, Supplemental Fig. S2*E*, and Supplemental Table S2). On average, 2227 and 2210 proteins were upregulated and downregulated in cancers, while 5622 and 3598 mRNAs were upregulated and downregulated in cancers. An average of 979 (43.96% of proteome and 17.41% of transcriptome) genes were upregulated, and an average of 875 (39.59% of proteome and 24.32% of transcriptome) genes were downregulated in both omics (Fig. 2*C*).

We then performed functional enrichment analysis for UGs and DGs in each cancer type and found that pathways including E2F targets, EMT, Myc targets, G2/M checkpoint, retinoblastoma gene in cancer, DNA replication, spliceosome, and mRNA processing were enriched. Many of these pathways were enriched across multiple cancer types, suggesting that a core set of pathways are associated with the occurrence and development of cancer (Fig. 2*E* and Supplemental Fig. S2*F*). Notably, mRNA splicing–related proteins were upregulated in cancers such as COAD, ESCC, LIHC, LUAD, LUSC, and OV (Supplemental Fig. S2*G*), indicating that splicing dysregulation is a characteristic in these cancers. Proteins involved in interferon-associated pathways, including type I and II interferon pathways, were specifically highly expressed in BRCA, ESCC, GBM, HNSC, KIRC, and PAAD (Supplemental Fig. S2, *H* and *I*), suggesting that a more immune-active tumor microenvironment in these cancers and possibly a better response to immunotherapy (68, 69). Conversely, proteins associated with fatty acid omega- and beta-oxidation, including both saturated and unsaturated fatty acid, were significantly downregulated in BRCA, COAD, HNSC, KIRC, LIHC, PAAD, and STAD (Supplemental Fig. S2*J*), suggesting that the accumulation of lipids and increase of energy requirements occur in these cancers (70). In BRCA, COAD, ESCC, HNSC, LIHC, LUAD, LUSC, and OV, the activity of the complement and coagulation cascade pathway, particularly at the level of complement activation, was reduced. Notably, genes involved in this pathway, including the classical pathway, alternative pathway, or lectin pathway, were significant downregulated across these cancers (Supplemental Fig. S2, *K* and *L*).

We then analyzed genes that are upregulated or downregulated at both RNA and protein levels in multiple cancer types (n ≥ 8), which were defined as PCUGs and PCDGs, respectively (Fig. 2, *F* and *G*, and Supplemental Table S3). There were 117 PCUGs and 102 PCDGs, accounting for only 1% of the coding genes. In particular, 33 oncogenes were found in the list of PCUGs, including some of the well-known oncogenes, such as MKI67, TRIP13, SMC4, UBE2C, CDK1, and TOP2A (Fig. 2*F* and Supplemental Table S3). It should also be noted that eight oncogenes were unexpectedly found in the list of PUDGs (Fig. 2*G* and Supplemental Table S3).

We further explored the biological functions of these PCUGs and PCDGs, finding that they are primarily associated with the aggressive phenotypes of cancers, such as cell cycle, metabolism of nucleotide, regulation of extracellular matrix, actomyosin structure organization, carbon dioxide transport, complement and coagulation, metabolism of amino acid, and regulation of system process (Supplemental Fig. S2*M*). We then explored potential therapeutic strategies that might target multiple cancer types based on PCUGs and PCDGs. The top 20 inhibitors with the highest potential for therapeutic purposes are targeting cyclin-dependent kinase (CDK), MEK, JAK, PI3K, ALK, histone deacetylase (HDAC), MTOR, and Topoisomerase. Interestingly, selumetinib (MEK inhibitor), vorinostat (HDAC inhibitor), crizotinib (ALK inhibitor), and palbociclib (CDK inhibitor) have already been successfully applied to treat patients with cancer, such as neurofibromatosis, lymphoma, multiple myeloma, and HER2-positive breast cancer (Fig. 2*H*).

### RRM2 and ADH1B Were Identified as Potential Pan-Cancer Diagnostic Markers

**Fig. 3:**
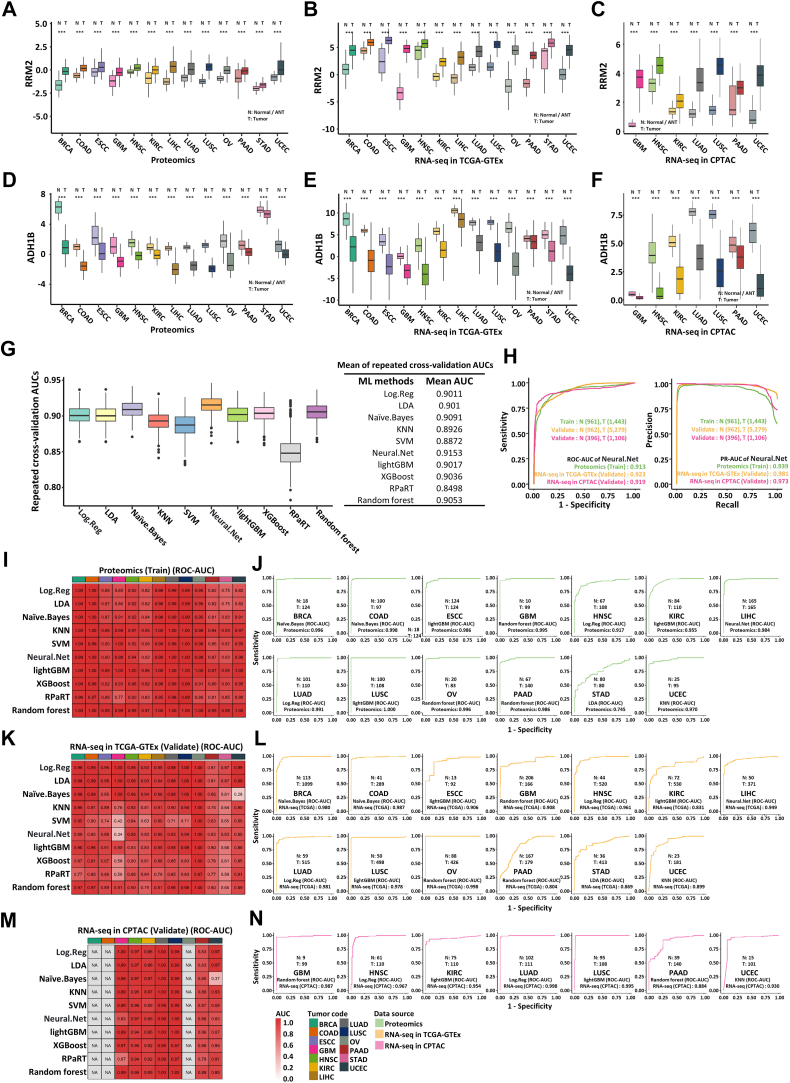
**RRM2 and ADH1B were identified as potential pan-cancer diagnostic markers**. *A*–*F*, the expression of RRM2 (*A*–*C*) and ADH1B (*D*–*F*) in proteomics data sourced from CPTAC (*A* and *D*) and transcriptomics data sourced from TCGA-GTEx (*B* and *E*) and CPTAC (*C* and *F*) is shown by boxplot. Within each color group, normal or ANTs are represented by lighter shades, while tumor tissues by darker shades. *p*-value was calculated by two-sided wilcoxon rank-sum test. The middle bar represents the median, and the box represents the interquartile range. Bars extend to 1.5 × the interquartile range (∗*p* < 0.05, ∗∗*p* < 0.01, ∗∗∗*p* < 0.001). *G*, boxplot (*left panel*) of ROC-AUCs calculated by 10 machine learning algorithms (Log.Reg, LDA, Naïve.Bayes, KNN, SVM, Neural.Net, lightGBM, XGBoost, RPaRT, and Random forest) with 100 rounds of five-fold cross-validation (CV) based on RRM2 and ADH1B z-score value in proteomic data. Table (*right panel*) of final ROC-AUCs calculated by 10 machine learning algorithms. *H*, ROC (receiver operating characteristic) curve (*left panel*) and PR (precision-recall) curve (*right panel*) of the Neural.Net model constructed by proteomic z-score value of RRM2 and ADH1B in all cancer type. *Green*, *yellow*, and *red* represent proteomic data in CPTAC (training cohort), RNA-seq in TCGA-GTEx (testing cohort), and RNA-seq in CPTAC (testing cohort), respectively. *I*, *K*, and *M*, heatmap of CPTAC proteomic (*I*), TCGA-GTEx transcriptomic (*K*), and CPTAC transcriptomic (*M*) ROC-AUCs of the prediction model constructed by 10 machine learning algorithms with 100 rounds of three-fold CV based on proteomic z-score value of RRM2 and ADH1B in each cancer type. *J*, *L*, and *N*, CPTAC proteomic (*J*), TCGA-GTEx transcriptomic (*L*), and CPTAC transcriptomic (*N*) ROC curve of the prediction model constructed by best learning algorithms in (*J*).

As shown in Figure 2*F*, ribonucleotide reductase regulatory subunit RRM2 is highly expressed, whereas alcohol dehydrogenase ADH1B is underexpressed across all 13 cancer types. RRM2 is implicated in the progression of various cancers, including breast cancer, lung cancer, liver cancer, kidney cancer, glioma, and pancreatic cancer, among others (71, 72, 73, 74, 75, 76, 77, 78, 79, 80, 81). Inhibition of RRM2 can overcome sunitinib-resistance in renal cell carcinoma (82). ADH1B catalyzes the conversion of ethanol to acetaldehyde in ethanol (alcohol) metabolism. Single nucleotide polymorphisms of ADH1B are associated with the risk of alcohol-related cancers, such as esophageal cancer, head and neck squamous cell cancer, liver cancer, stomach cancer, and colorectal cancer (83, 84, 85, 86). ADH1B has also been reported to suppress cell proliferation in pancreatic cancer and colorectal cancer (87, 88). The aberrant expression of RRM2 and ADH1B across all 13 cancer types prompted us to explore whether they can server as pan-cancer diagnostic markers to distinguish between normal and tumor samples. The magnitude of differential expression of RRM2 and ADH1B in each cancer type was presented based on proteomic and transcriptomic data sourced from CPTAC as well as transcriptomic data from TCGA-GTEx (Fig. 3, *A*–*F*). Considering the low specificity and sensitivity of existing or even the lack of molecular diagnostic markers in many cancers (89, 90, 91, 92, 93), we next explored the feasibility of applying this pair of genes as diagnostic biomarkers to distinguish between normal and tumor samples for pan-cancer. By standardizing the abundance levels in gene direction, the abundance distribution patterns of RRM2 and ADH1B became more consistent across the different datasets (Supplemental Fig. S3, *A* and *B*). The proteomic sourced from CPTAC, including all non-tumor and tumor samples, were used as the training cohort. Ten machine learning algorithms with 100 rounds of five-fold CV were then employed to calculate the AUC of ROC curve. Using ROC-AUC value as the criterion, the Neural network (Neural.Net) algorithm showed the best predictive performance for tumor samples among all 10 algorithms, with CV-AUCs of 0.915 in proteomic data from CPTAC (Fig. 3*G*). Subsequently, transcriptomic data from TCGA-GTEx and CPTAC were used as validation cohorts; ROC-AUCs and precision recall-AUCs were both greater than 0.9, demonstrating the strong diagnostic capabilities of the prediction model (Fig. 3*H*). Similarly, the CV-AUCs were 0.947 when transcriptomic data from TCGA-GTEx was used as the training cohort (Supplemental Fig. S3*C*). In this case, proteomic and transcriptomic data from CPTAC were served as validation cohorts; ROC-AUCs and precision recall-AUCs were both greater than 0.88 (Supplemental Fig. S3*D*). Besides, diagnostic models for each cancer type were constructed and validated in the same way. Both RRM2 and ADH1B showed excellent diagnostic performance across all cancer types tested except STAD and PAAD (Fig. 3, *I*–*N* and Supplemental Fig. S3, *E*–*V*), supporting the potential of this pair of genes as pan-cancer diagnostic biomarkers to distinguish between normal and tumor tissues. Furthermore, a diagnostic model based on RRM2 and ADH1B was incorporated into a web tool to aid researchers and clinicians in cancer diagnosis (http://www.cppa.site/sysproteome/predict). Next, the prognostic value of RRM2 and ADH1B was also explored in cancer. Notably, high expression of RRM2 was correlated with poor prognosis, while that of ADH1B was linked to favorable prognosis in LIHC (Supplemental Fig. S3, *W*–*Z*).

### Dysregulated Genes and Pathways in Different Tumor Stages/Cancer Progression

**Fig. 4:**
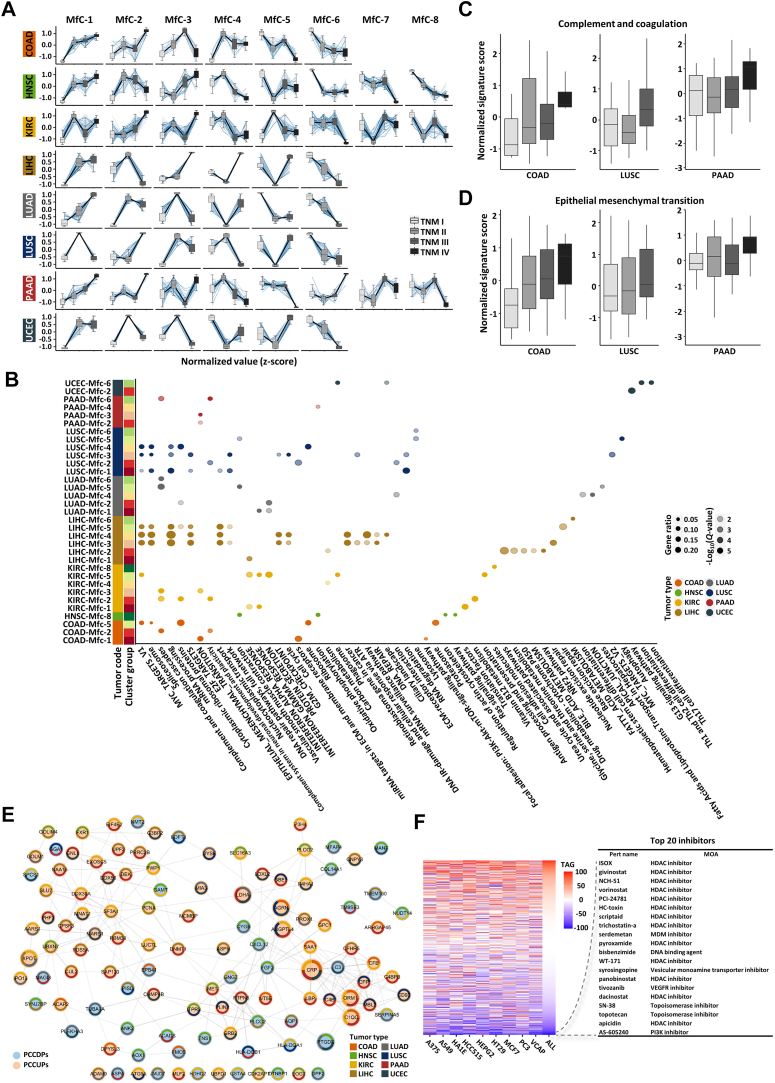
**Dysregulated genes and pathways during cancer progression**. *A*, the expression trend analysis of differentially expressed proteins during cancer progression (TNM stage) in each cancer type. The trend clusters were ranked in the order from continuous increase to continuous decline in abundance. When cancer samples in TNM stage IV were included, the number of trend clusters is specified as 8. Otherwise, the number of clusters is specified as 6. *B*, the dot plot was used to display significance of enrichment (by over representation analysis method in R package clusterProfiler) for gene sets from Hallmark, KEGG, and wikiPathway with proteins in each trend cluster in each cancer type. The *Q* value of the gene set must be less than 0.05, and the number of intersections must be at least 5. The different colors represent different cancer types, the size of the points indicates the proportion of input proteins present in the functional gene set, and the shade of color represents the negative log_10_(*Q* value). *C* and *D*, boxplot of signature score of complement and coagulation pathway (C) and epithelial mesenchymal transformation (EMT) process (*D*) at different TNM stages of COAD, LUSC, and PAAD. Signature score was calculated by ssGSEA algorithm. The four different shades of *gray* represent TNM stages I, II, III, and IV, respectively. *E*, protein–protein interaction networks of pan-cancer continuously upregulated and downregulated proteins (PCCUPs and PCCDPs). *Red* and *blue* circles represent PCCUPs and PCCDPs, respectively. *F*, the workflow of drug prediction. The PCCUPs (n = 75) and PCCDPs (n = 39) were used as the query signature to match the reference profiles of perturbagens in CMap to calculate connectivity scores. Perturbagens are sorted by connectivity score in increasing order, and the *top* perturbagens are predicted as candidate drugs.

The TNM stage is a widely used classification system in cancer pathology and clinical medicine. T, N, and M represent the primary site of the tumor (tumor), the involvement of lymph nodes (node), and the presence of distant metastasis (metastasis), respectively. Unlike independent molecular subtyping systems for different cancer types, the TNM stage system provides a unified evaluation standard for most cancers. To investigate the molecular and biological pathways disrupted in early and advanced cancer stages, we used TNM stage as an indicator of cancer progression and compiled TNM stage information for all 13 cancer types from the proteomic samples (Supplemental Fig. S4*A*). To ensure the accuracy of the analysis, cancer types with desultory TNM stages or with a limited number of samples in TNM stages were excluded. Eventually, eight cancer types (COAD, HNSC, KIRC, LIHC, LUAD, LUSC, PAAD, and UCEC) were retained. Differential expression analysis was conducted for these eight cancer types by grouping samples according to TNM stage. The results showed that COAD exhibited the lowest number of differentially expressed proteins (371 proteins), while KIRC displayed the highest number of differentially expressed proteins (1188 proteins) (Supplemental Fig. S4*B*). Based on the differentially expressed proteins, the expression trend analysis was performed in each cancer type according to TNM stage. The trend clusters were ranked in the order from continuous increase to continuous decrease in abundance (Fig. 4*A* and Supplemental Fig. S4*C*). Subsequently, functional enrichment analysis was conducted for each trend cluster within each cancer type (Fig. 4*B*). We found that the proteins involved in the complement and coagulation pathway and EMT pathway changed consistently in COAD, LUSC, and PAAD, with the abundance of the related proteins increases as cancer progresses (Fig. 4, *C* and *D*, and Supplemental Fig. S4*D*). This was the most prominent in COAD. Some studies have reported that complement proteins can promote invasion and metastasis in multiple cancer types through various mechanisms (94, 95, 96, 97, 98, 99). The expression profiles of representative proteins involved in these two pathways in COAD, LUSC, and PAAD were displayed in the form of boxplots and heatmaps (Supplemental Fig. S4, *E*–*J*). In contrast, the trend analysis also revealed that the abundance of proteins related to fatty acid and lipoprotein transport decreased with the progression of LUAD (Supplemental Fig. S4*K*), the abundance of oxidative phosphorylation-related proteins decreased with the progression of HNSC (Supplemental Fig. S4*L*), and the abundance of cilia-related proteins decreased with the progression of UCEC (Supplemental Fig. S4*M*). In addition, pathways, such as immune-related pathway and DNA damage repair pathway, also exhibited alterations with the progression in different cancers (Fig. 4*B*). The expression trend clustering is based on fuzzy clustering algorithm, which is not suitable for integration analysis. Therefore, only proteomic data were used to reveal the dysregulated events in cancer progression.

We then identified continuously up-regulated and down-regulated proteins in different tumor stage (Supplemental Fig. S4, *N* and *O*). Furthermore, we identified 75 and 39 proteins that showed a continuously upregulated and downregulated trend, respectively, in at least two cancer types. We defined these two group proteins as PCCUPs and PCCDPs in TNM stages (Fig. 4*E*, Supplemental Fig. S4, *P* and *Q*, and Supplemental Table S4). Eight proteins (C-reactive protein, AGRN, XPOT, LDHA, C1QC, ORM1, ANGPTL4, and prostaglandin D2 synthase [PTGDS]) exhibited a continuously upward or downward trend in three or more cancer types. Notably, C-reactive protein showed continuous upregulation in the progression of COAD, KIRC, PAAD, and UCEC, while PTGDS showed continuous downregulation in the progression of UCEC, HNSC, and LUAD (Supplemental Fig. S4, *P* and *Q*). Functional enrichment analysis revealed that PCCUPs were primarily associated with complement and coagulation cascades, mismatch repair, HIF1 signaling pathway, and EMT, among others. On the other hand, PCCDPs were mainly related to fatty acid metabolism and amino acid metabolism, among others (Supplemental Fig. S1, *R* and *S*). Based on these findings, we explored potential therapeutic strategies based on PCCUPs and PCCDPs. Inhibitors targeting HDAC, MDM, VEGFR, PI3K, and Topoisomerase were identified as potential drug candidates for interfering the progression of multiple cancer types (Supplemental Fig. S4, *R* and *S*).

### Establishment and Validation of Tumor Stage Classification Models

**Fig. 5:**
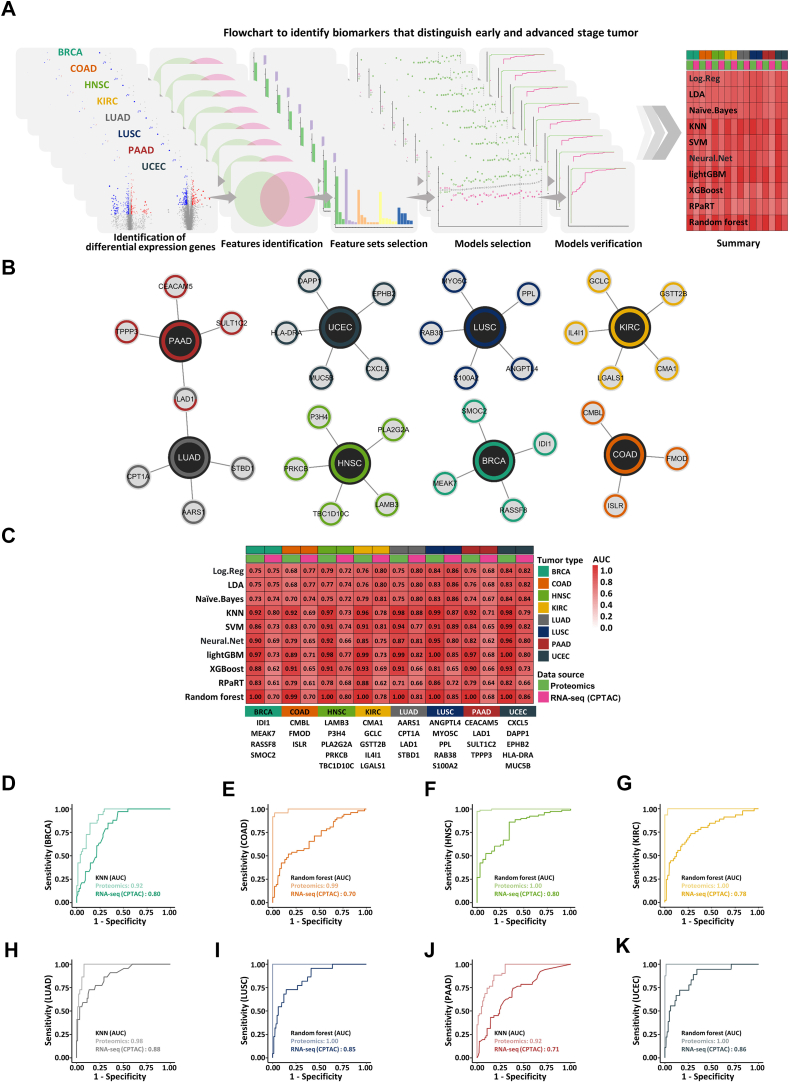
**Establishment and validation of tumor stage classification models**. *A*, the workflow for the construction of tumor stage classification models: screening of differential expressed mRNAs and proteins, feature identification, feature set selection, model selection, and model validation. *B*, network visualization of genes included in the stage classification gene panels. Gene nodes are colored according to cancer types. *C*, heatmap of ROC-AUC values calculated by the optimal stage classification model for each cancer type. *Green* and *red* colors represent ROC-AUC values for proteomic data and transcriptomic data sourced from CPTAC, respectively. *D*–*K*, ROC curve of the optimal stage classification model in in BRCA (*D*), COAD (*E*), HNSC (*F*), KIRC (*G*), LUAD (*H*), LUSC (*I*), PAAD (*J*), and UCEC (*K*). *Green* and *red* colors represent ROC-AUCs for proteomic and transcriptomic data sourced from CPTAC, respectively.

One of the most important goals in cancer precision medicine is to accurately distinguish the pathological stage of tumors for facilitating effective therapeutic interventions to improve patient survival. Therefore, we next sought to differentiate between early- and advanced-stage cancer patients at the molecular level, aiming for more precise treatments. We utilized proteomic data along with corresponding transcriptomic data from CPTAC, ensuring consistent clinical information across samples. Each cancer sample was classified according to its TNM stage, in which TNM I and II stages were classified as early stages, while TNM III and IV stages as advanced stages (Supplemental Fig. S5, *A* and *B*). To ensure sufficient sample size for robust analysis, cancer types with inadequate sample number at each stage were excluded, and then eight cancer types (BRCA, COAD, HNSC, KIRC, LUAD, LUSC, PAAD, and UCEC) were retained. We then constructed stage classification models for each cancer type following a standardized pipeline, which includes five steps: differential protein and mRNA screening, feature identification, feature set selection, model selection, and model validation (Fig. 5*A*). Based on the differential analysis results of both proteomic and transcriptomic datasets, genes that showed differential expression at both protein and mRNA levels were selected as input for model construction (Supplemental Fig. S5, *C* and *D*). Considering that clinical classifiers need to be as stable as possible with a minimal number of features, SVM algorithms were employed to construct the model based on proteomic data during feature set selection. The ROC-AUC value was used as the evaluation criterion, and the maximum feature number was set as 1, 2, 3, 4, and 5 for 100 rounds of repeated sampling verification. Then, feature sets with the top five frequencies at each maximum feature number were chosen as the candidate feature sets (Supplemental Fig. S5, *E*–*L*). During the model selection phase, 10 machine learning algorithms were combined with the candidate feature sets to identify the top 10 model decided by ROC-AUCs (Supplemental Fig. S5, *M*–*T*). Finally, taking the number of features, stability of model, and prediction performance into consideration, the optimal stage classification biomarkers and models based on proteomic data for each cancer type were selected (Fig. 5, *B*–*K*). Through this pipeline, stage classification biomarker panels were established for eight cancer types, comprising a total of 34 genes (Fig. 5*B* and Supplemental Fig. S5, *U*–*Z*’’). The ROC-AUCs for proteomic data all exceeded 0.9, and the ROC-AUCs for transcriptomic data were all above 0.7 (Fig. 5, *D*–*K*). Considering the inherent heterogeneity of tumor samples and the relatively small molecular differences between early and advanced tumor tissues, we believe that the performance of the stage classification models is sufficient to meet the needs of clinical practice. Based on these findings, the optimal classification model for each cancer type was incorporated into a web tool (http://www.cppa.site/sysproteome/predict), enabling researchers and clinicians to readily classify tumors as early or advanced stage. Moreover, we also attempted to identify pan-cancer biomarkers for pathological stage classification, but failed to do so.

### Establishment and Validation of Prognostic Risk Stratification Models

**Fig. 6:**
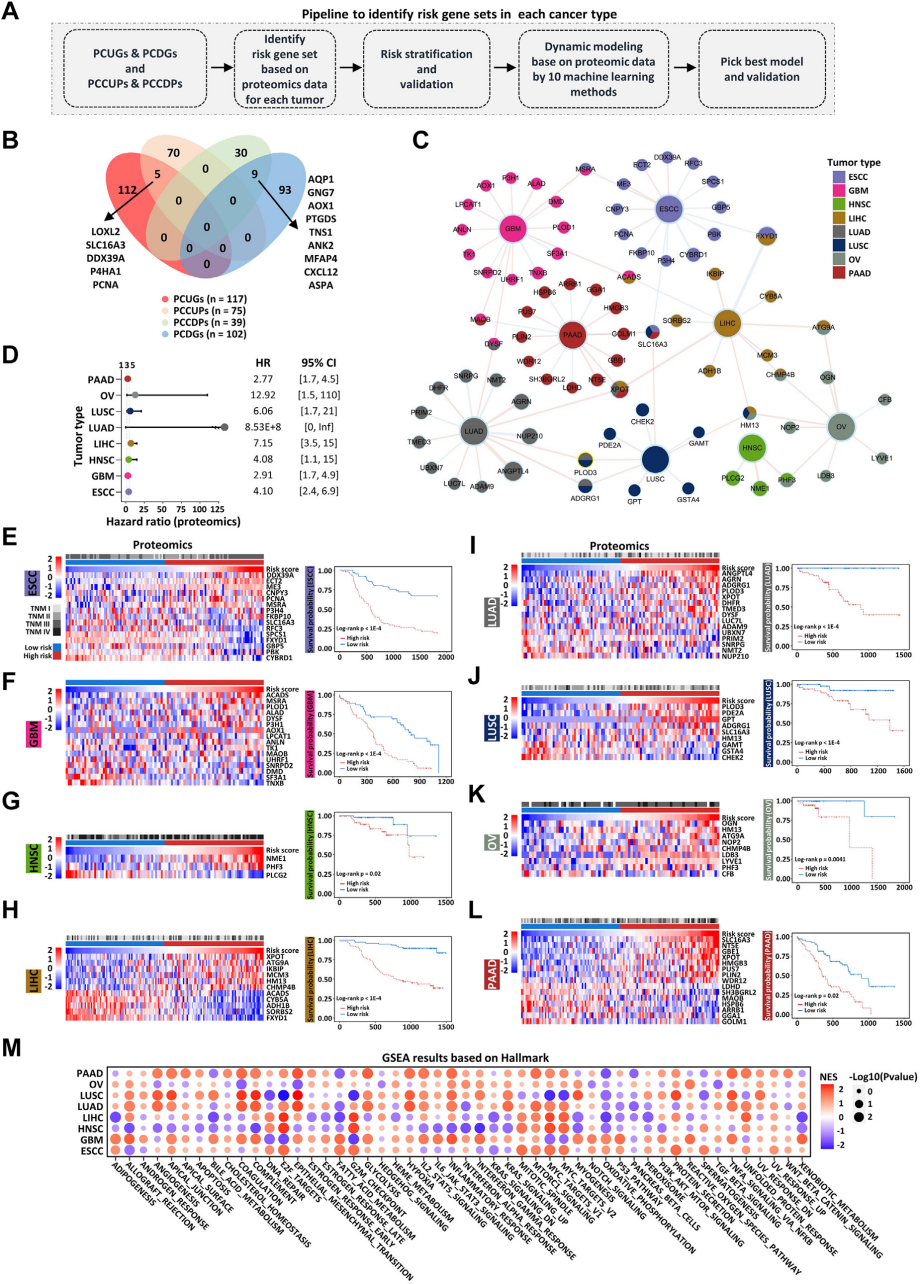
**Establishment and validation of prognostic risk stratification model.***A*, the workflow for the construction of prognostic risk stratification models for each cancer type: integration of 4 pan-cancer–related gene sets, identify risk gene sets in proteomic data for each cancer type, risk stratification, and validation, dynamic modeling based on proteomic data, and pick best model and validation. *B*, the intersection of PCUGs, PCDGs, PCCUPs, and PCCDPs was shown by Venn diagram. *Red*, *blue*, *orange*, and *green* colors represent PCUGs, PCDGs, PCCUPs, and PCCDPs, respectively. *C*, network visualization of genes included in the prognostic risk stratification models. Gene nodes are colored according to cancer types. *D*, the hazard ratios (HRs) of high-risk groups of all eight cancer types in proteomic data are shown by forest plot (*left panel*), and the 95 confidence intervals (CIs) of corresponding HRs are shown by a table (*right panel*). *E*–*L*, the abundance of risk proteins involved in prognostic risk stratification models is shown by heatmap (*left panel*) and the prognosis of high-risk group in ESCC (*E*), GBM (*F*), HNSC (*G*), LIHC (*H*), LUAD (*I*), LUSC (*J*), OV (*K*), and PAAD (*L*) is shown by Kaplan-Meier plot (*right panel*). *M*, normalized enrichment score (NES) (by ssGSEA method in R package clusterProfiler) for gene sets from Hallmark gene sets with differential scores between high- and low-risk group of all proteins in each cancer type is shown by dot plot. The different colors represent NES and the size of the points indicates negative log_10_(*Q* value).

Within the realm of cancer precision medicine, one of the paramount objectives is to improve patient survival rates. Accurately predicting a patient's survival time serves as a crucial indicator for guiding effective drug treatment decisions. To this end, we sought to construct a prognostic risk stratification model based on the following methodology (Fig. 6*A*). Due to the absence or the limited survival information for certain cancer types, eight cancer types (ESCC, GBM, HNSC, LIHC, LUAD, LUSC, OV, and PAAD) were retained for subsequent analysis. As described above, we successfully defined four pan-cancer gene sets, PCUGs and PCDGs representing pan-cancer dysregulated genes between tumor and non-cancer samples, as well as PCCUPs and PCCDPs representing pan-cancer dysregulated proteins in different cancer stages (Figs. 2, *F*, and *G*, and 4*E*). Interestingly, five genes (LOXL2, SLC16A3, DDX39A, P4HA1, and PCNA) were present in both PCUGs and PCCUPs, and nine genes (AQP1, GNG7, AOX1, PTGDS, TNS1, ANK2, MFAP4, CXCL12, and ASPA) appeared in both PCDGs and PCCDPs, suggesting that these genes are involved in both the occurrence and development of cancer (Fig. 6*B*). Among these gene sets, proteomic data was used as training cohort and LASSO-Cox algorithm was utilized to screen the prognostic risk genes and calculate their corresponding risk coefficients for each cancer type (Supplemental Fig. S6, *A*–*H* and Supplemental Table S5). Then, a network diagram was constructed to visualize the prognostic risk genes across all eight cancer types (Fig. 6*C*). The risk score of each cancer sample was calculated by summing the product of the risk coefficient and the abundance of each risk gene. Subsequently, tumor samples were categorized into high- and low-risk groups according to the median value of the risk score. Additionally, transcriptomic data sourced from TCGA-GTEx and CPTAC were used as validation cohorts, and then tumor samples in these two cohorts were classified into high- and low-risk groups based on the same risk genes determined by proteomic data. Within the proteomic data, the hazard ratios of high-risk group exceeded two for each cancer type, indicating that high-risk group patients exhibit a significantly higher risk of mortality than low-risk patients (Fig. 6*D*), which was confirmed by the hazard ratios of high-risk group from transcriptomic data (Supplemental Fig. S6, *I* and *J*). Furthermore, the abundance of risk proteins in the high- and low-risk groups was visualized in the form of heatmap (Fig. 6, *E*–*L*). Survival analysis revealed that patients in the high-risk group exhibited significantly worse overall survival with log-rank *p* value below 0.05 (Fig. 6, *E*–*L*). Notably, these findings were supported by the transcriptomic data sourced from CPTAC (Supplemental Fig. S6, *K*–*P*) as well as TCGA-GTEx (Supplemental Fig. S6, *Q*–*X*), such that the patients in the high-risk group were associated with poor survival outcomes. GSEA analysis results revealed that proteins in the high-risk group was mainly enriched with Myc target, MTORC1 signaling, Complement, and Inflammatory response (Fig. 6*M*). We also tried to identify pan-cancer risk proteins for prognostic risk stratification, but failed to do so.

Given that the risk stratification system is independent of the TNM stages, we further explored whether integrating this system with the TNM stages could enhance the accuracy for predicting the prognosis of cancer patients. Time-dependent ROC analysis demonstrated that, at any time frame ranging from 1 to 5 years, integrating both systems significantly outperformed either one alone in predicting patient prognosis (Supplemental Fig. S7, *A*–*H*).

Due to the complexity and potential biases involved in risk group stratification, which requires the integration of all risk gene abundances and their corresponding risk coefficients, along with categorization based on a fixed median value. Therefore, we optimized this process through machine learning methods. Ten machine learning algorithms were employed to learn expression pattern of risk proteins for each cancer type, and then constructed models were validated by transcriptomic datasets (Supplemental Fig. S8, *A*–*H*). Subsequently, the most effective prognostic risk stratification models were selected for each cancer type (Supplemental Fig. S9, *A*–*I*). The ROC curves demonstrated that these models can effectively predict high- and low-risk patients, regardless of whether the risk gene abundance data were derived from proteomic or transcriptomic data (Supplemental Fig. S9, *B*–*I*). Similarly, the optimal prognostic risk stratification model for each cancer type has been built into a web tool, enabling researchers and clinicians to make informed prognostic risk judgments for cancer patients (http://www.cppa.site/sysproteome/predict).

## Discussion

The investigation of genomics and transcriptomics is the first step towards precision oncology, and proteomics has, in many quarters, been considered the next logical step in expanding our understanding of tumor biology because it provides information that complements genomic and transcriptomic data (18). However, due to the limitations of MS technology, proteomics has lagged behind in terms of quantitative accuracy and sensitivity compared to transcriptomics (100, 101, 102, 103, 104). Therefore, we assembled a comprehensive dataset comprising transcriptomic and proteomic data from 13 different types of cancer, including tens of thousands of patient samples.

In these datasets, we observed that the majority of genes exhibit decent correlations at mRNA and protein level, with only approximately 3% showing inverse correlations. Notably, the genes that are highly correlated in expression at protein and mRNA level are primarily associated with pathways related to cell-cell junction, actin filament assembly, and amino acid catabolic process. Furthermore, we observed that the expression of tissue-specific genes is underpresented in cancer, indicating that tumor tissues have departed from their original tissue characteristics, evolving into new structural entities. Previous studies have primarily focused on cancer tissues alone (35, 36, 37), whereas our analysis includes both normal tissues/ANTs and tumor tissues, leading us to discover that some certain signaling pathways, such as splicing, interferon response, fatty acid oxidation, and complement activation, are disordered in tumors across multiple cancer types when compared to non-tumor samples. Moreover, genes that are upregulated and downregulated at both RNA and protein levels across multiple types of cancers were identified and defined as PCUGs and PCDGs, respectively, which encompass a large number of oncogenes such as MKI67, TRIP13, SMC4, UBE2C, CDK1, and TOP2A. Drug prediction based on PCUGs and PCDGs revealed that several approved drugs (selumetinib, vorinostat, crizotinib, and palbociclib) for specific cancer types might be effective as pan-cancer treatments, similar to the widely used pan-cancer drugs like the PD-1 inhibitor Pembrolizumab (Keytruda), which is approved for any solid tumor with high microsatellite instability or mismatch repair deficiency, and larotrectinib (Vitrakvi), which is approved for solid tumors with neurotrophic receptor tyrosine kinase gene fusion. Among these inhibitors, Purvalanol A, a small molecule targeting the CDK family, predicted the most promising therapeutic responses. CDKs have been widely recognized for their critical role in promoting malignant behaviors in nearly all cancers (105, 106). Purvalanol A has also shown efficacy in inhibiting cell growth in various cancer types, including estrogen receptor–positive breast cancer, non-small cell lung cancer, and colorectal cancer (107, 108, 109, 110). Strikingly, we identified that RRM2 and ADH1B are upregulated and downregulated, respectively, in all 13 cancer types, suggesting that this pair of genes could serve as promising pan-cancer diagnostic biomarkers. The construction and validation of pan-cancer diagnostic models based on this pair of genes also supported this conclusion. These results have sparked interest in RRM2 and ADH1B as commercialized cancer diagnostic biomarkers. The overpresentation of RRM2 in tumor tissues also implies its critical biological function, raising questions about whether inhibitors targeting RRM2 could offer new avenues for pan-cancer treatment.

In addition to pan-cancer analysis between tumor and non-tumor samples, we also explored disordered events in the pathological progression of cancers. However, due to the incomplete information about TNM stages, the analysis was limited to eight cancer types. Our analysis revealed that the complement and coagulation pathways were abnormally interconnected with EMT pathway in COAD, LUSC, and PAAD. Some studies have shown the connection between these two pathways. For example, C3a has been reported to decrease the expression of E-cadherin to promote EMT in ovarian cancer (94). This raised the question of whether targeting key proteins in the complement system and EMT pathway could offer a promising strategy to delay tumor invasion and metastasis or even eradicate cancer cells. Additionally, we defined a group of proteins that are continuously upregulated and downregulated (PCCUPs and PCCDPs) during tumor progression in at least two cancer types, and drug prediction based on these proteins reveals that HDACs play a key role in cancer development. HDACs are crucial components in the epigenetic regulator protein family, which alter the transcription of oncogenes and tumor suppressor genes through removing acetyl modifications from histones, thereby affecting tumor initiation and progression. Furthermore, we constructed stage classification models to distinguish patients in advanced stages (TNM III and IV) from those in early stages (TNM I and II). However, due to the heterogeneity among tumor samples, validation based on transcriptomic data for COAD and PAAD yielded less satisfactory results. These findings underscore the complexity of cancer progression and the potential for novel treatment strategies by targeting specific biological pathways and key proteins involved in pan-cancer development.

The goal of cancer precision medicine extends beyond the mere diagnosis of cancer to encompass the assessment of tumor malignancy and risk level, which is crucial for guiding appropriate treatment selection. To achieve this, we defined a group of prognostic risk genes using LASSO-Cox algorithms based on PCUGs, PCDGs, PCCUPs, and PCCDPs, among which XPOT, SLC16A3, HM13, ACADS, MSRA, FXYD1A, TG9A, PLOD3, ADGRG1, PHF3, MAOB, and DYSF were identified as risk genes in at least two different cancer types. Furthermore, we also established prognostic risk stratification models for different cancer types by machine learning and validated the reliability of prognostic risk stratification models in multiple datasets. However, due to the absence of survival information for some cancer types, identification of risk gene sets and construction of prognostic risk stratification models were only possible for eight cancer types. As more comprehensive survival data become available, we aim to expand our analysis to include additional cancer types and identify novel risk gene sets and construct corresponding prognostic risk stratification models. So far, we have identified biomarkers and constructed models for diagnosis, pathological classification, and prognostic risk stratification, which differs from previous pan-cancer proteomics researches for defining more detailed subtype of pan-cancer, such as proteome-based subtypes (42, 111) or immune-based subtypes (37). Together, these efforts aim to advance the pan-cancer research field by facilitating the identification of commonalities and specificities across cancers.

In summary, our study investigated the dysregulated genes and biological pathways during the occurrence and development of pan-cancer and defined four pan-cancer–related gene sets, PCUGs and PCDGs representing genes upregulated and downregulated, respectively, in pan-cancer, and PCCUPs and PCCDPs representing proteins continuously upregulated and downregulated, respectively, along with cancer development. Additionally, we developed web-based tools for pan-cancer diagnostic models, stage classification models, and prognostic risk stratification models. Furthermore, we predicted the inhibitors that could potentially be used for cancer therapy.

## Data Availability

All data used in this study are publicly available. Proteomic data are available from CPTAC and PRIDE. Transcriptomic data sourced from CPTAC and TCGA are available at the Genomic Data Commons (https://gdc.cancer.gov/). Transcriptomic data sourced from GTEx are available at the GTEx portal (https://www.gtexportal.org/).

## Supplemental data

This article contains supplemental data.

## Conflict of Interest

The authors declare no competing interests.
