# Supplementary material for: Integrated Analysis of Proteome and Transcriptome Profiling Reveals Pan-Cancer-Associated Pathways and Molecular Biomarkers
Source: Mol Cell Proteomics. 2025 Jan 28;24(3):100919. doi: 10.1016/j.mcpro.2025.100919 (PMC11907456; doi:10.1016/j.mcpro.2025.100919)
Supplement: Supplementary_Figures [file mmc6.pdf]

A

Proteins quantifiable at different cutoff

Dataset	Proteins	Description
Prot1	16623	Proteins quantifiable in at least one sample
Prot2	10755	Proteins quantifiable in at least 1/4 samples
Prot3	9059	Proteins quantifiable in at least 1/2 samples
Prot4	7510	Proteins quantifiable in at least 3/4 samples
Prot5	3630	Proteins quantifiable in all samples

B

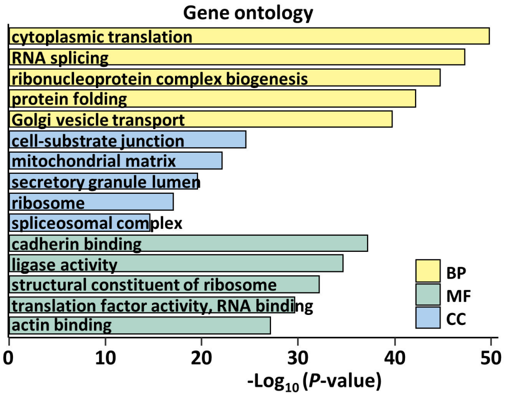

C D E F

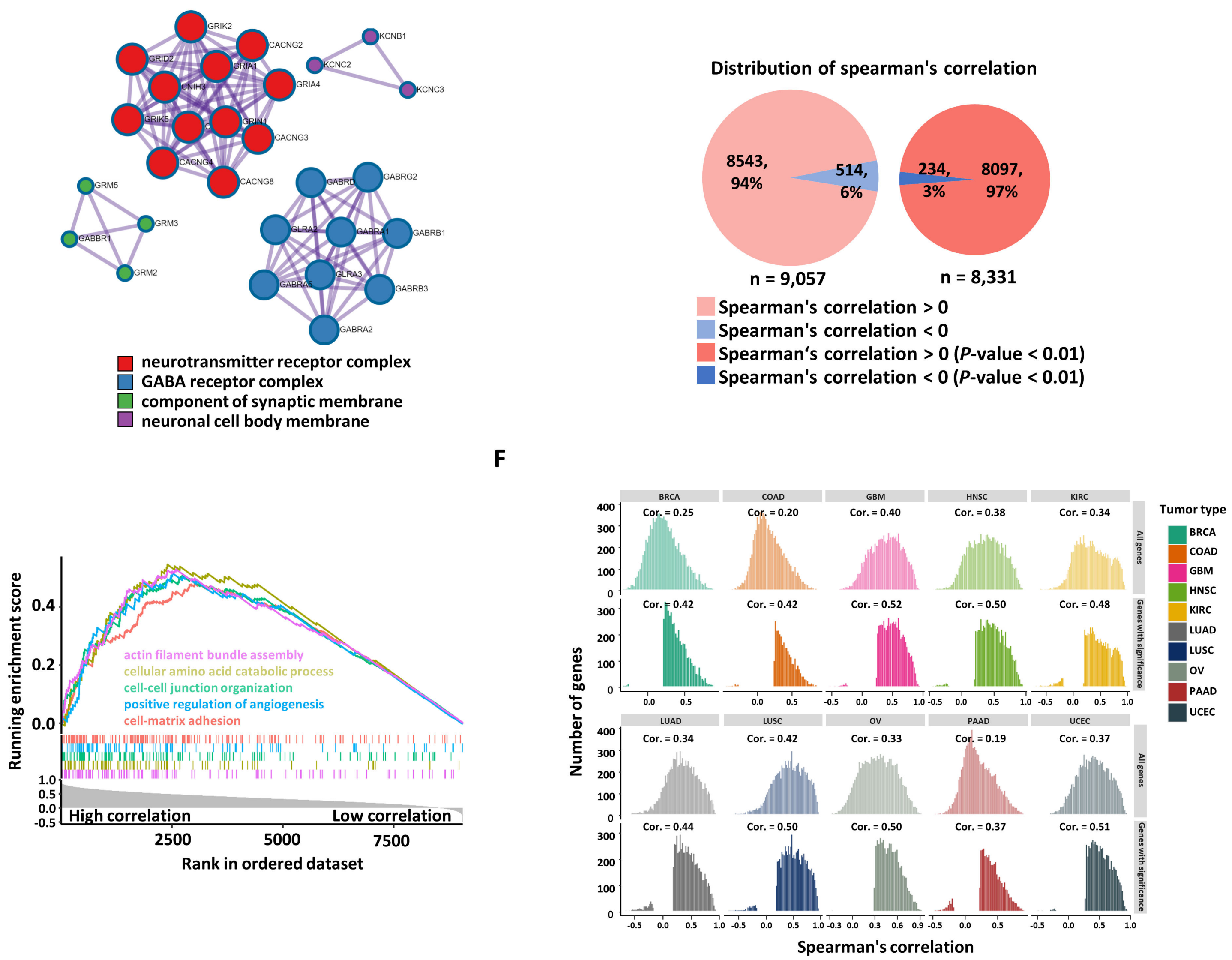

Figure S1

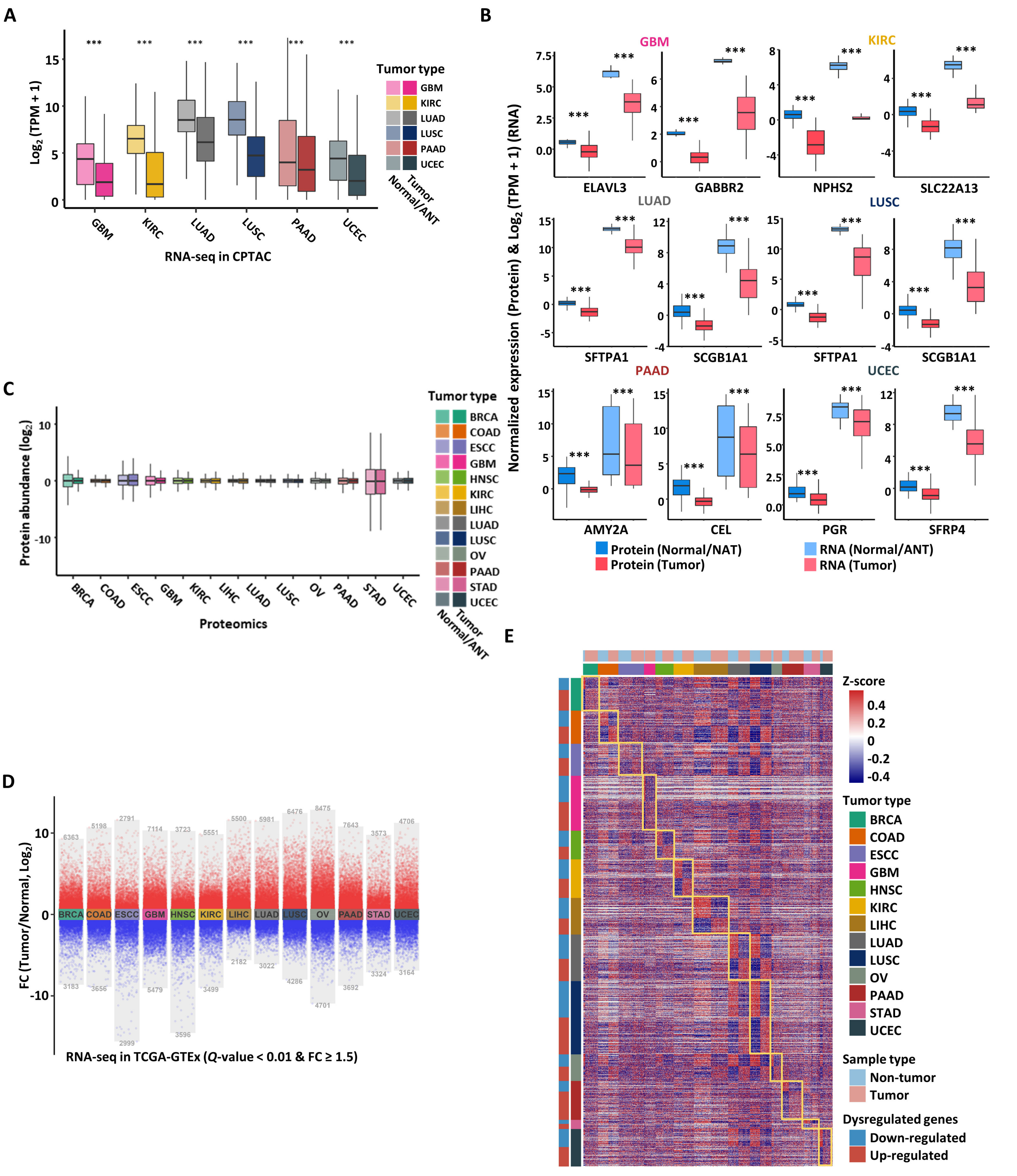
A
B
C
D
E
Tumor type
Normal/ANT
Tumor
RNA-seq in CPTAC
Log2 (TPM + 1)
GBM
KIRC
LUAD
LUSC
PAAD
UCEC
STAD
BRCA
COAD
ESCC
HNSC
LIHC
OV
Normalized expression (Protein) & Log2 (TPM + 1) (RNA)
ELAVL3
GABBR2
NPHS2
SLC22A13
SFTPA1
SCGB1A1
AMY2A
CEL
PGR
SFRP4
Protein (Normal/NAT)
Protein (Tumor)
RNA (Normal/ANT)
RNA (Tumor)
Protein abundance (log2)
Proteomics
FC (Tumor/Normal, Log2)
RNA-seq in TCGA-GTEx (Q-value < 0.01 & FC ≥ 1.5)
Z-score
Sample type
Non-tumor
Dysregulated genes
Down-regulated
Up-regulated

Figure S2

F

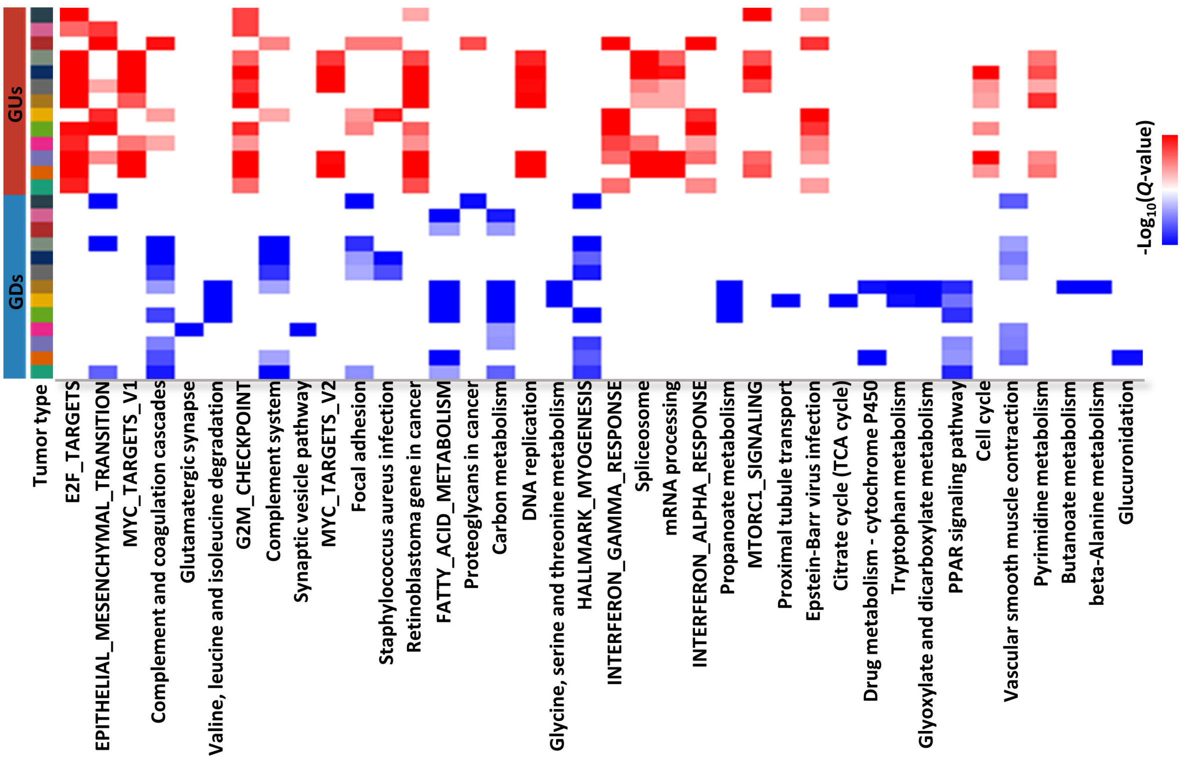

G

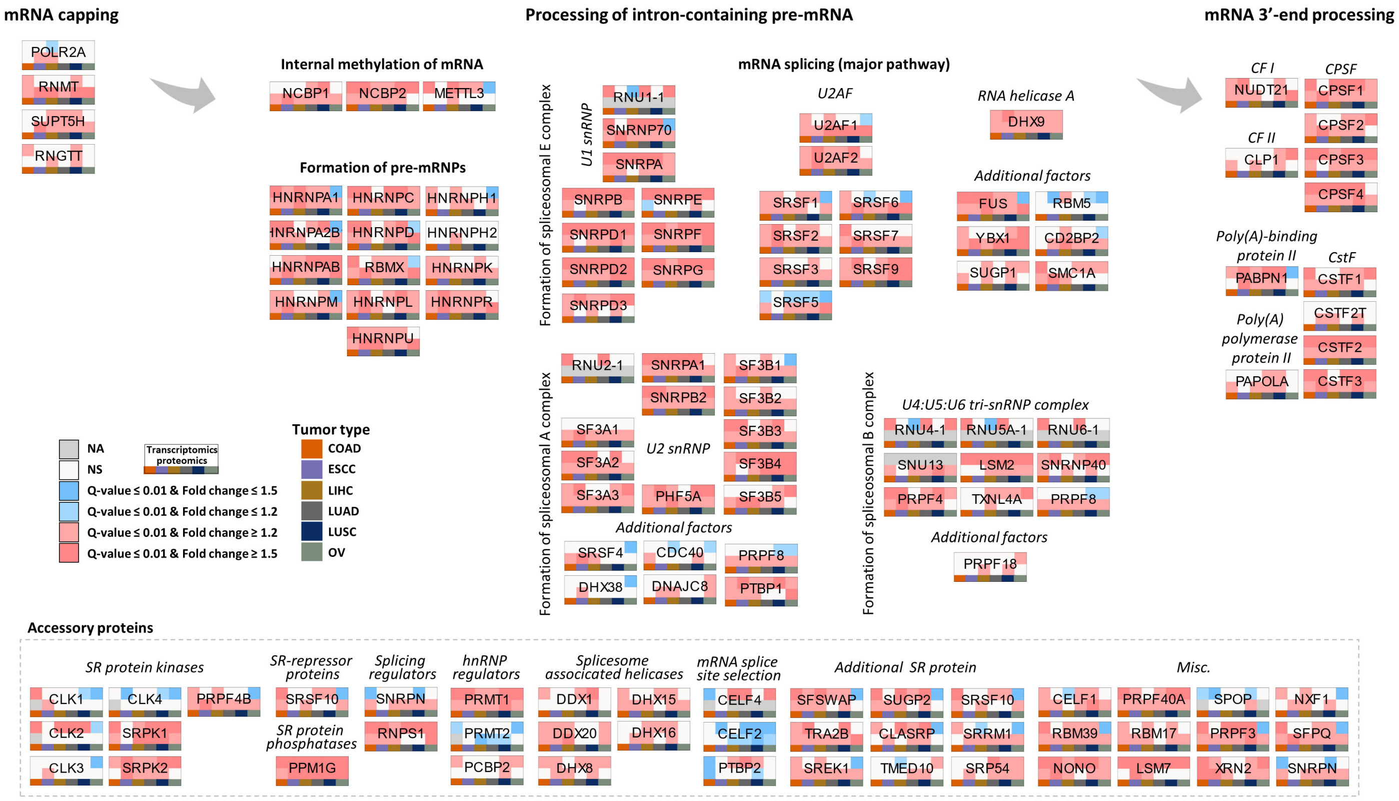

Figure S2 cont'd

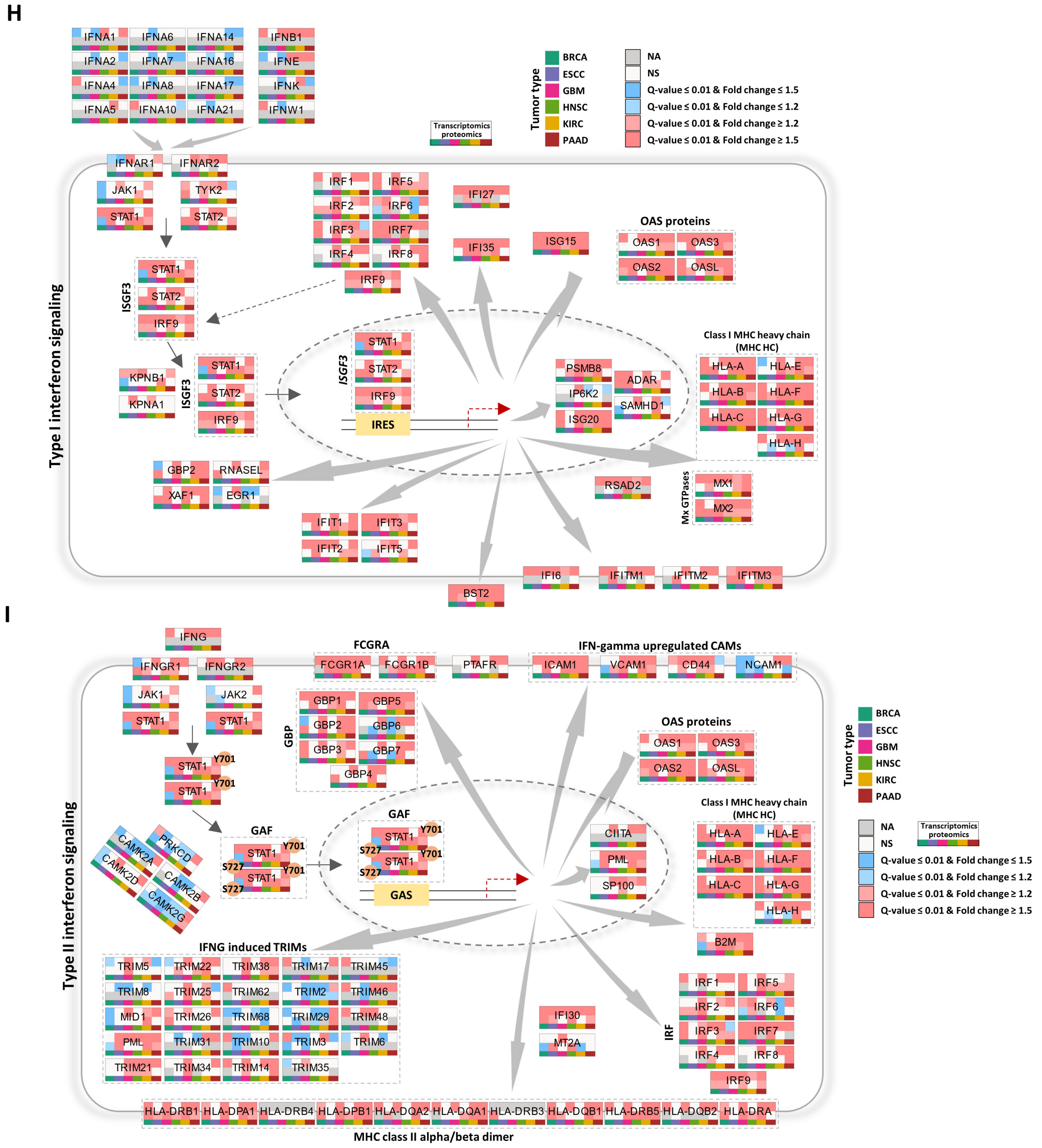

Figure S2 cont'd

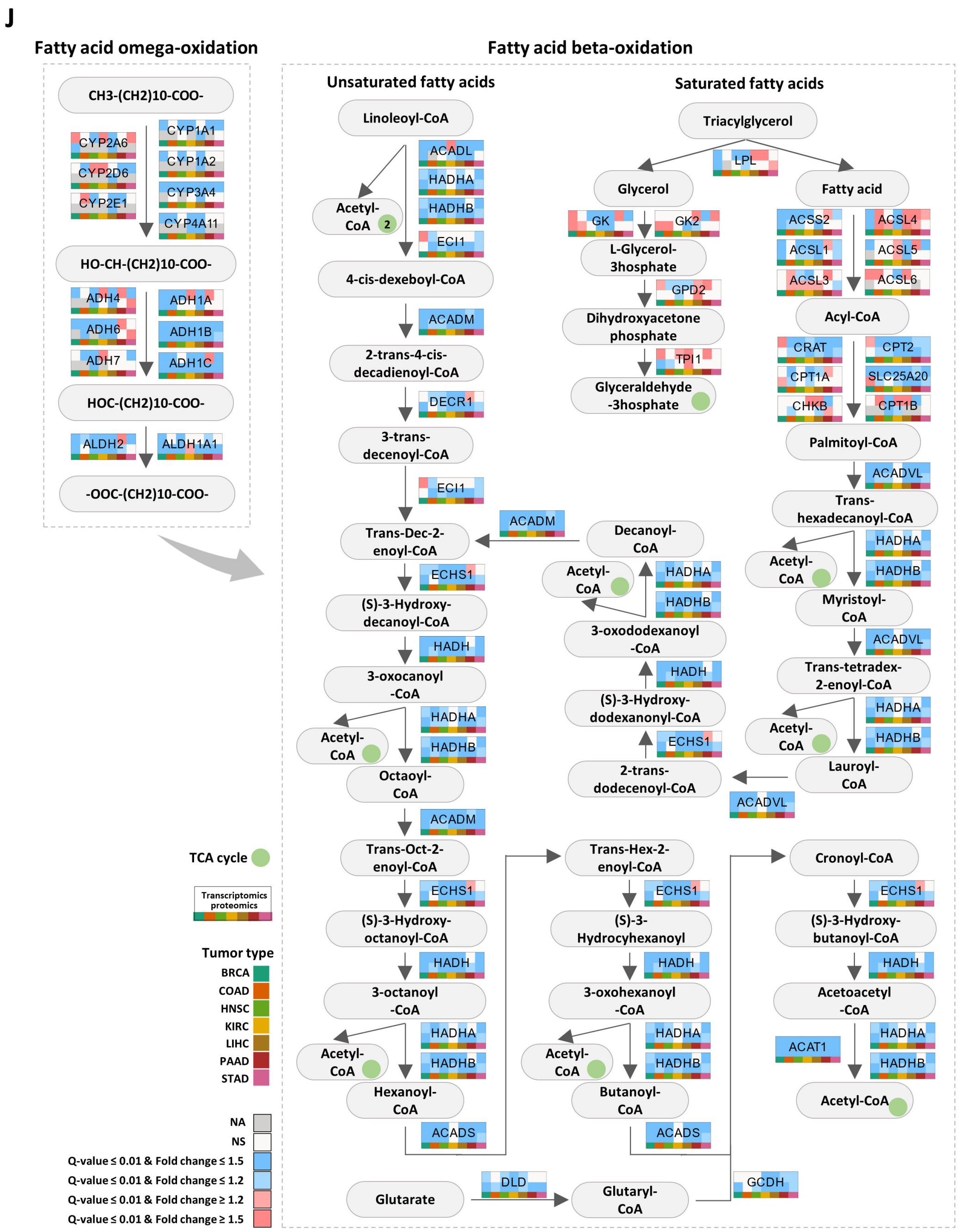
J
Fatty acid omega-oxidation
Fatty acid beta-oxidation
CH3-(CH2)10-COO-
CYP2A6
CYP2D6
CYP2E1
CYP1A1
CYP1A2
CYP3A4
CYP4A11
HO-CH-(CH2)10-COO-
ADH4
ADH6
ADH7
ADH1A
ADH1B
ADH1C
HOC-(CH2)10-COO-
ALDH2
ALDH1A1
-OOC-(CH2)10-COO-
Unsaturated fatty acids
Saturated fatty acids
Linoleoyl-CoA
ACADL
HADHA
HADHB
Acetyl-CoA
2
ECI1
4-cis-dexeboyl-CoA
ACADM
2-trans-4-cis-decadienoyl-CoA
DECR1
3-trans-decenoyl-CoA
ECI1
Trans-Dec-2-enoyl-CoA
ACADM
Decanoyl-CoA
ECHS1
(S)-3-Hydroxy-decanoyl-CoA
HADH
3-oxocanoyl-CoA
HADHA
HADHB
Acetyl-CoA
Octaoyl-CoA
ACADM
Trans-Oct-2-enoyl-CoA
ECHS1
(S)-3-Hydroxy-octanoyl-CoA
HADH
3-octanoyl-CoA
HADHA
HADHB
Acetyl-CoA
Hexanoyl-CoA
ACADS
Glutarate
DLD
Glutaryl-CoA
GCDH
Triacylglycerol
LPL
Glycerol
Fatty acid
GK
GK2
L-Glycerol-3hosphate
GPD2
Dihydroxyacetone phosphate
TPI1
Glyceraldehyde-3hosphate
ACSS2
ACSL1
ACSL3
ACSL4
ACSL5
ACSL6
Acyl-CoA
CRAT
CPT1A
CHKB
CPT2
SLC25A20
CPT1B
Palmitoyl-CoA
ACADVL
Trans-hexadecanoyl-CoA
HADHA
HADHB
Acetyl-CoA
Myristoyl-CoA
ACADVL
Trans-tetradex-2-enoyl-CoA
HADHA
HADHB
Acetyl-CoA
Lauroyl-CoA
ACADVL
2-trans-dodecenoyl-CoA
ECHS1
(S)-3-Hydroxy-dodexanonyl-CoA
HADH
3-oxododexanoyl-CoA
HADHA
HADHB
Acetyl-CoA
Trans-Hex-2-enoyl-CoA
ECHS1
(S)-3-Hydrocyhexanoyl
HADH
3-oxohexanoyl-CoA
HADHA
HADHB
Acetyl-CoA
Butanoyl-CoA
ACADS
Cronoyl-CoA
ECHS1
(S)-3-Hydroxy-butanoyl-CoA
HADH
Acetoacetyl-CoA
ACAT1
HADHA
HADHB
Acetyl-CoA
TCA cycle
Transcriptomics proteomics
Tumor type
BRCA
COAD
HNSC
KIRC
LIHC
PAAD
STAD
NA
NS
Q-value ≤ 0.01 & Fold change ≤ 1.5
Q-value ≤ 0.01 & Fold change ≤ 1.2
Q-value ≤ 0.01 & Fold change ≥ 1.2
Q-value ≤ 0.01 & Fold change ≥ 1.5

K

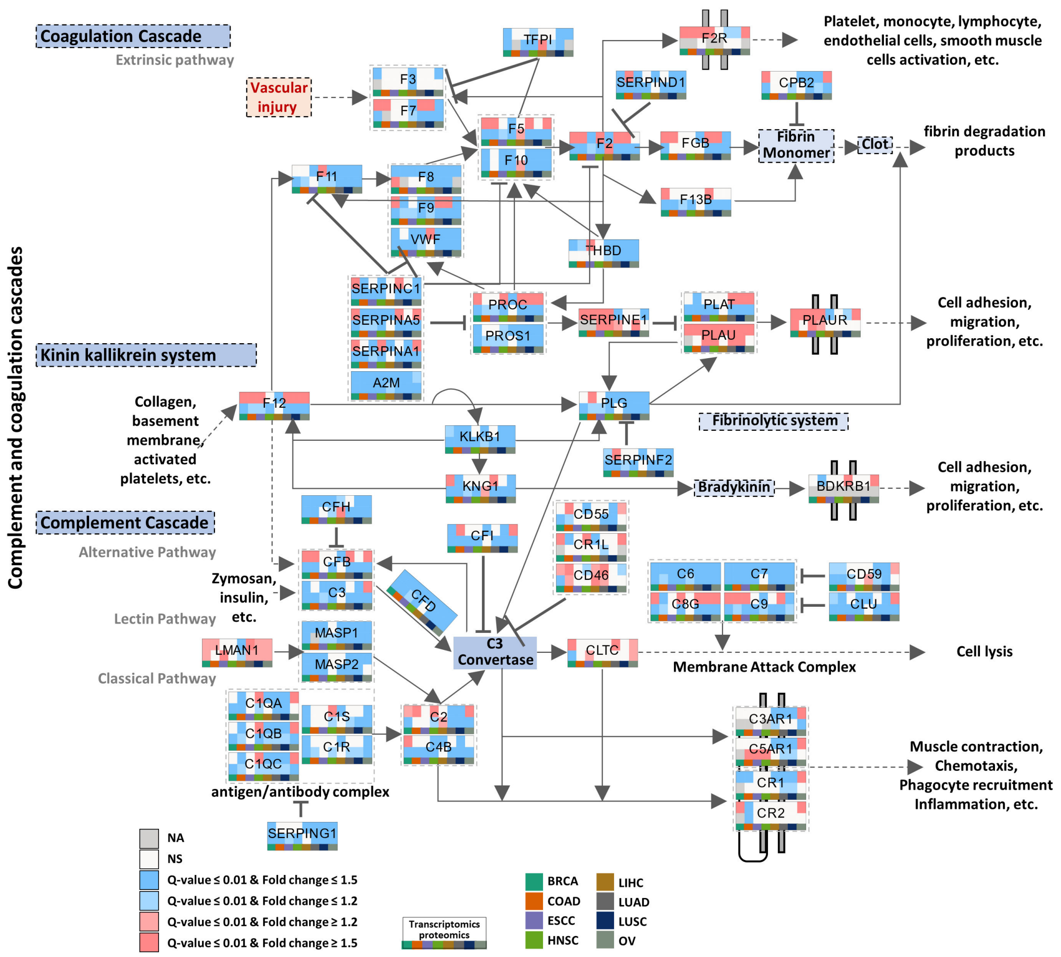

L

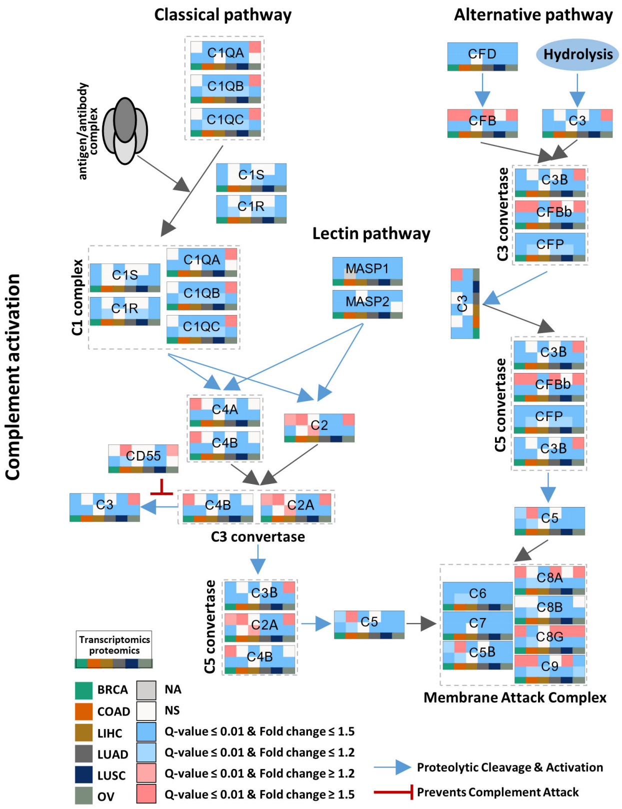

Figure S2 cont'd

M

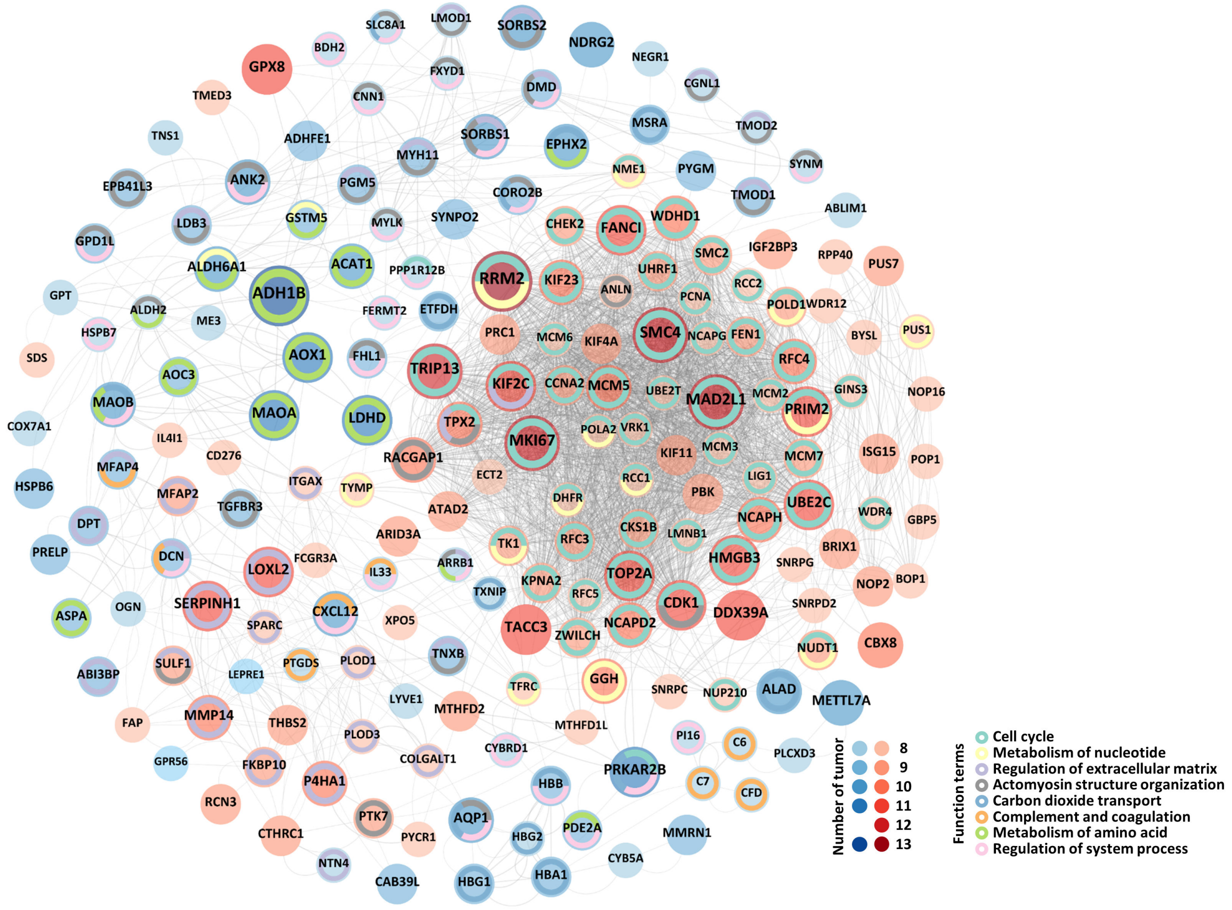

Figure S2 cont'd

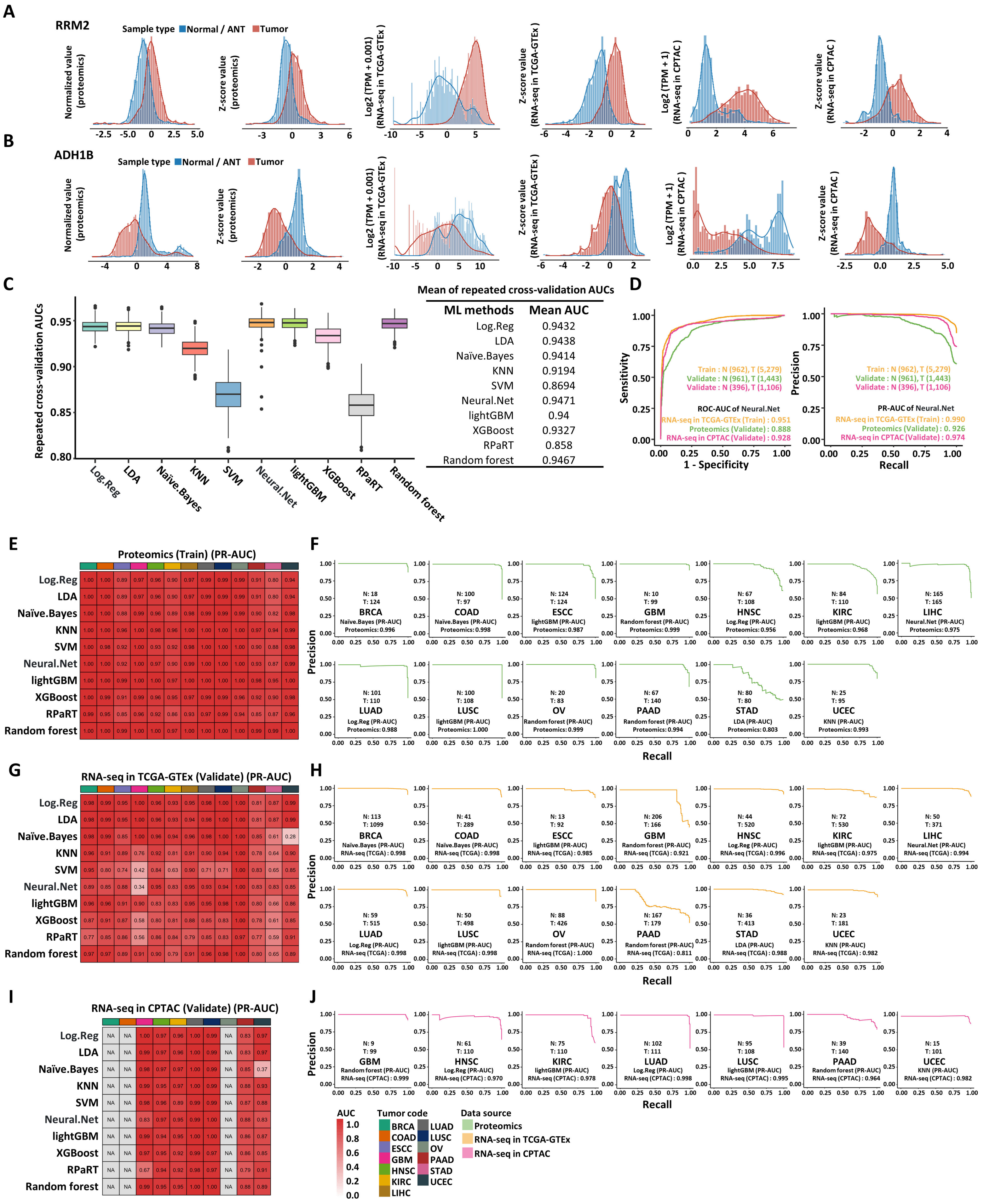
A
RRM2
Sample type Normal / ANT Tumor
Normalized value (proteomics)
Z-score value (proteomics)
Log2 (TPM + 0.001) (RNA-seq in TCGA-GTEx)
Z-score value (RNA-seq in TCGA-GTEx)
Log2 (TPM + 1) (RNA-seq in CPTAC)
Z-score value (RNA-seq in CPTAC)
B
ADH1B
Sample type Normal / ANT Tumor
C
Repeated cross-validation AUCs
Log.Reg
LDA
Naïve.Bayes
KNN
SVM
Neural.Net
lightGBM
XGBoost
RPaRT
Random forest
Mean of repeated cross-validation AUCs
ML methods Mean AUC
Log.Reg 0.9432
LDA 0.9438
Naïve.Bayes 0.9414
KNN 0.9194
SVM 0.8694
Neural.Net 0.9471
lightGBM 0.94
XGBoost 0.9327
RPaRT 0.858
Random forest 0.9467
D
Sensitivity
1 - Specificity
Train : N (962), T (5,279)
Validate : N (961), T (1,443)
Validate : N (396), T (1,106)
ROC-AUC of Neural.Net
RNA-seq in TCGA-GTEx (Train) : 0.951
Proteomics (Validate) : 0.888
RNA-seq in CPTAC (Validate) : 0.928
Precision
Recall
PR-AUC of Neural.Net
RNA-seq in TCGA-GTEx (Train) : 0.990
Proteomics (Validate) : 0. 926
RNA-seq in CPTAC (Validate) : 0.974
E
Proteomics (Train) (PR-AUC)
F
G
RNA-seq in TCGA-GTEx (Validate) (PR-AUC)
H
I
RNA-seq in CPTAC (Validate) (PR-AUC)
J
AUC
Tumor code
BRCA
COAD
ESCC
GBM
HNSC
KIRC
LIHC
LUAD
LUSC
OV
PAAD
STAD
UCEC
Data source
Proteomics
RNA-seq in TCGA-GTEx
RNA-seq in CPTAC

Figure S3

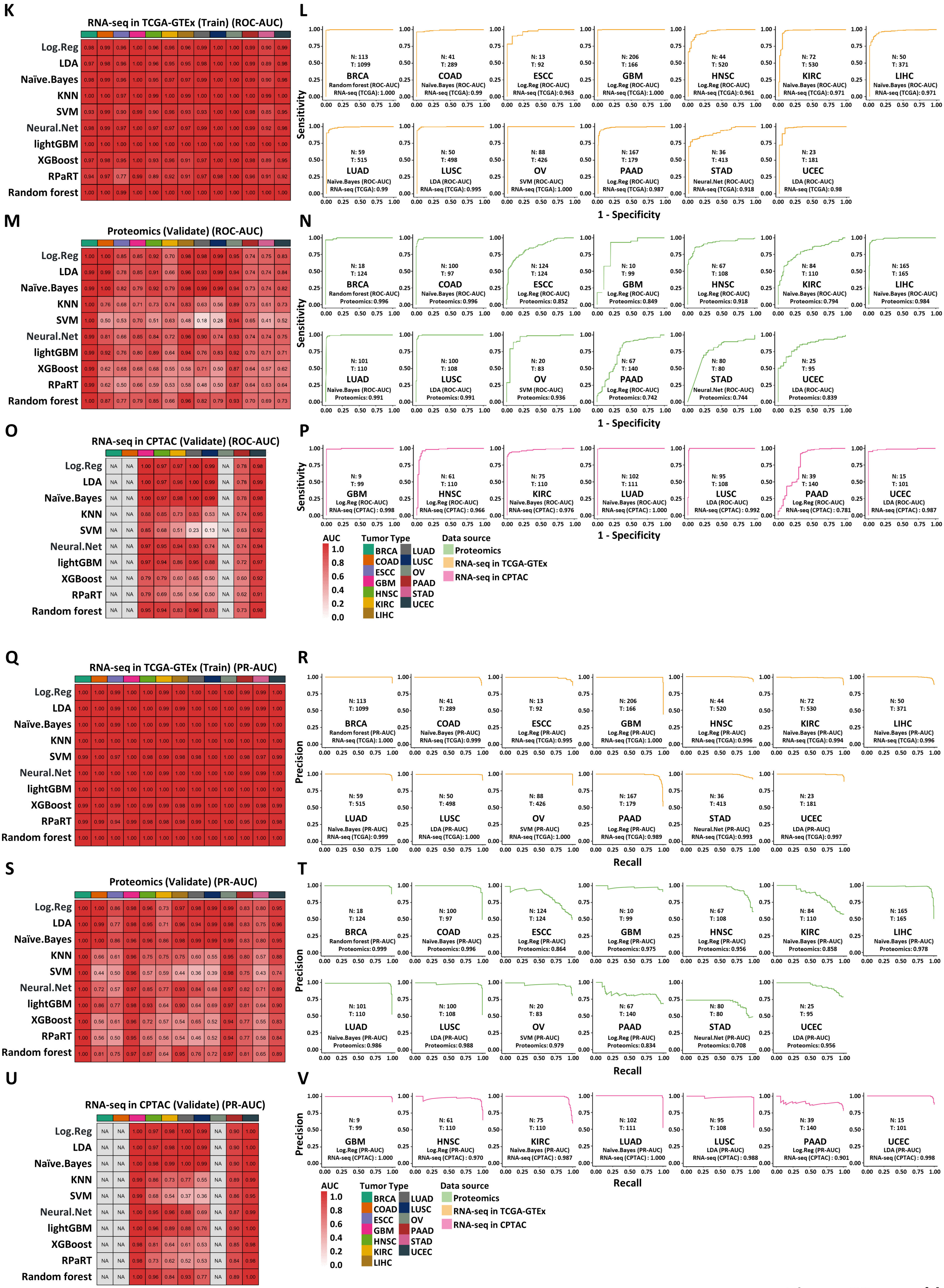

Figure S3 cont'd

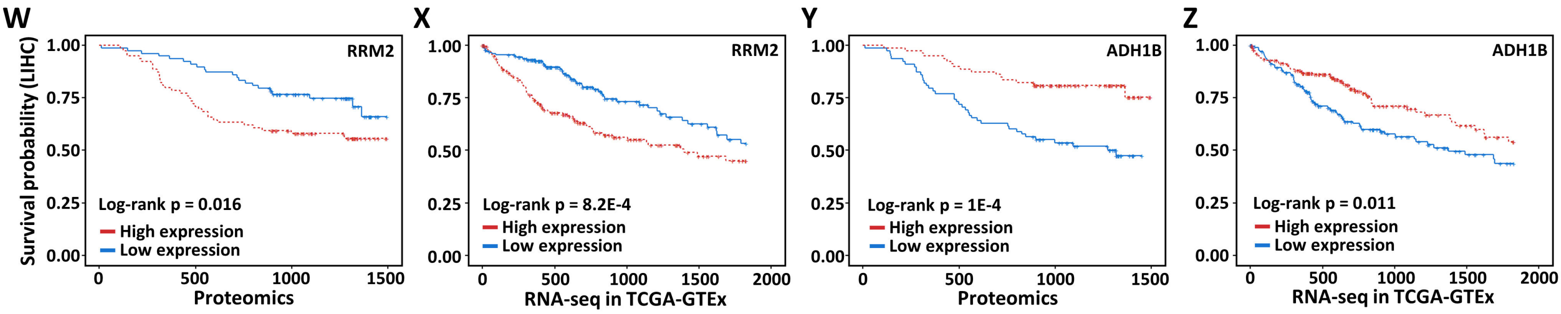

Figure S3 cont'd

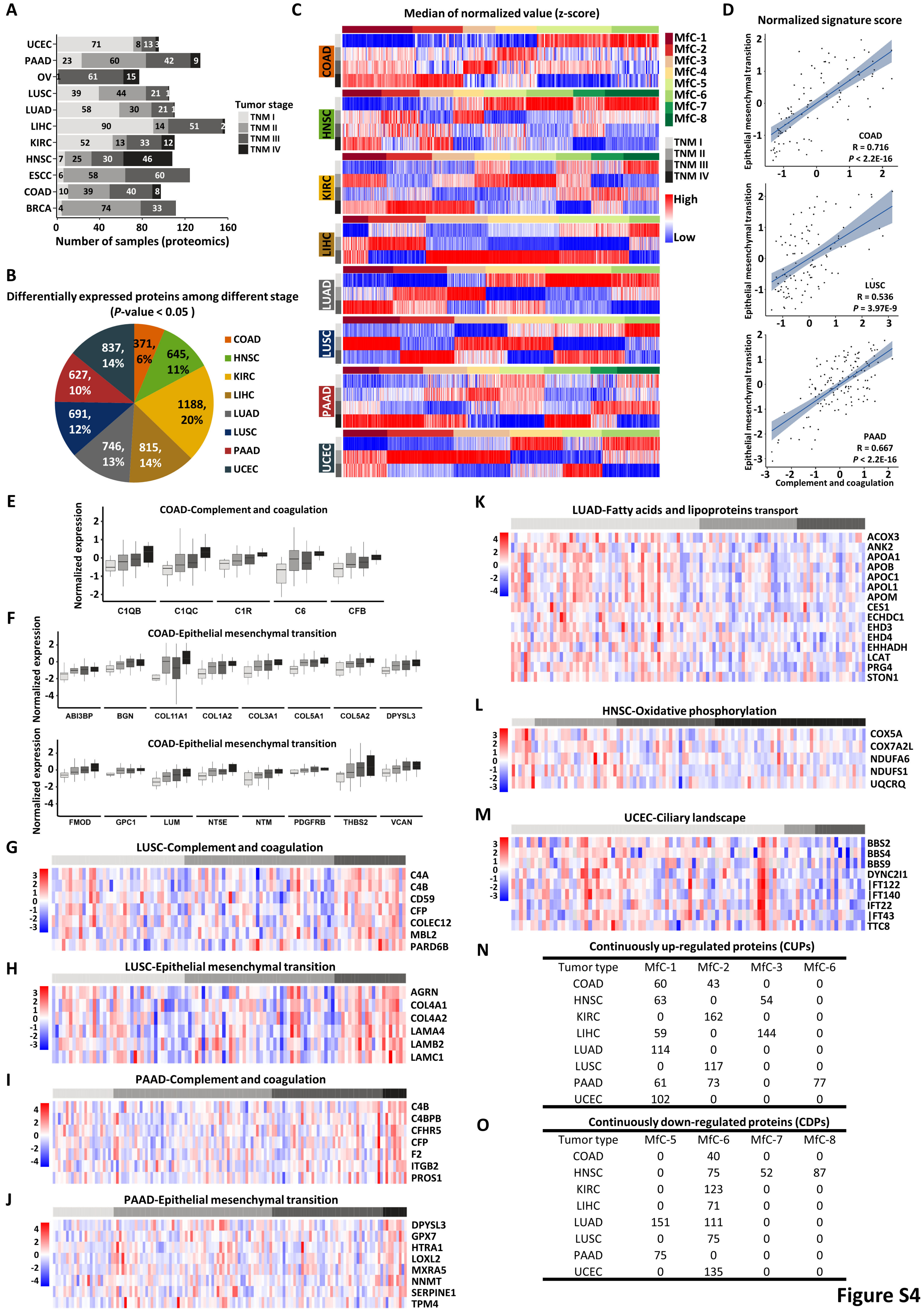

N

Continuously up-regulated proteins (CUPs)

Tumor type	MfC-1	MfC-2	MfC-3	MfC-6
COAD	60	43	0	0
HNSC	63	0	54	0
KIRC	0	162	0	0
LIHC	59	0	144	0
LUAD	114	0	0	0
LUSC	0	117	0	0
PAAD	61	73	0	77
UCEC	102	0	0	0

O

Continuously down-regulated proteins (CDPs)

Tumor type	MfC-5	MfC-6	MfC-7	MfC-8
COAD	0	40	0	0
HNSC	0	75	52	87
KIRC	0	123	0	0
LIHC	0	71	0	0
LUAD	151	111	0	0
LUSC	0	75	0	0
PAAD	75	0	0	0
UCEC	0	135	0	0

Figure S4

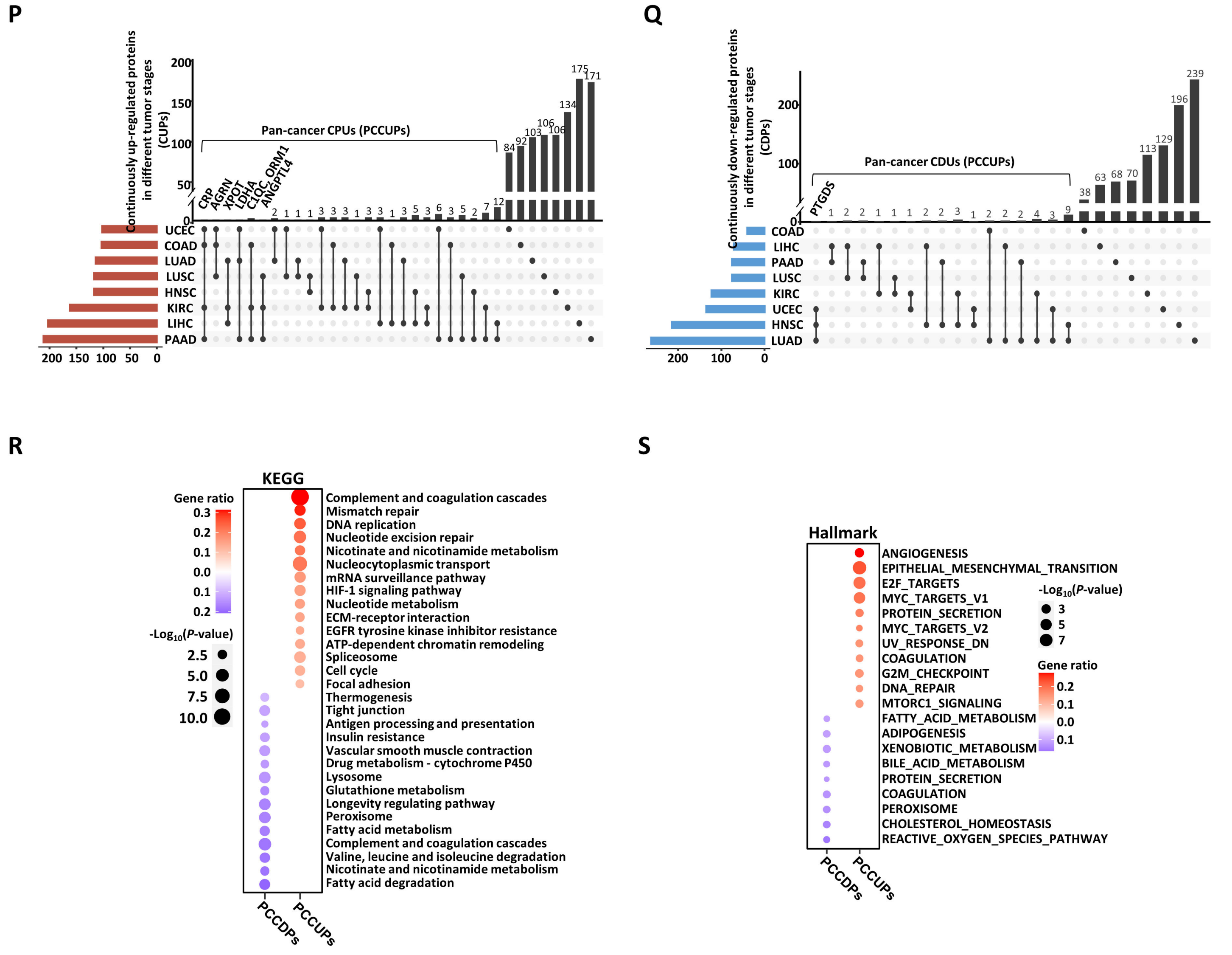

Figure S4 cont'd

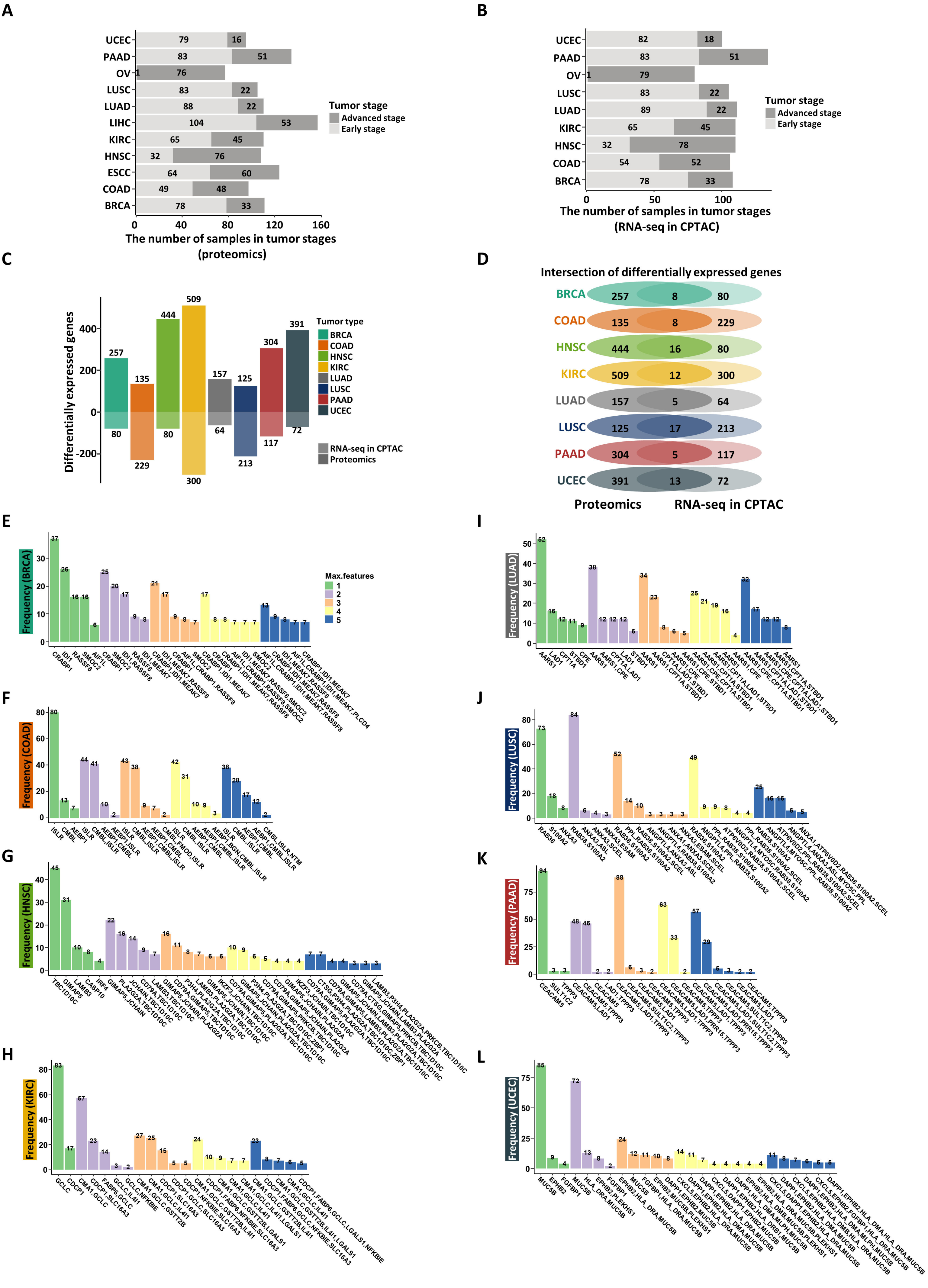
A
B
C
D
E
F
G
H
I
J
K
L
The number of samples in tumor stages (proteomics)
The number of samples in tumor stages (RNA-seq in CPTAC)
Tumor stage
Advanced stage
Early stage
Differentially expressed genes
Tumor type
BRCA
COAD
HNSC
KIRC
LUAD
LUSC
PAAD
UCEC
RNA-seq in CPTAC
Proteomics
Intersection of differentially expressed genes
Max.features
Frequency (BRCA)
Frequency (COAD)
Frequency (HNSC)
Frequency (KIRC)
Frequency (LUAD)
Frequency (LUSC)
Frequency (PAAD)
Frequency (UCEC)

Figure S5

M

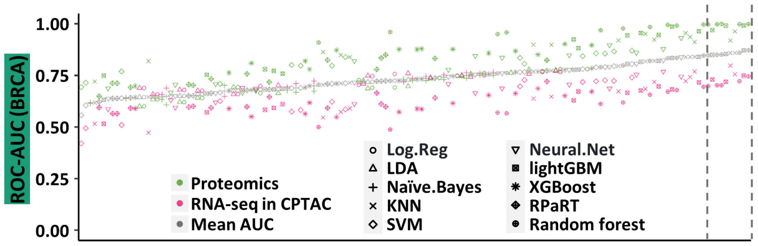

Top 10 Feature sets

Order	Feature set	MS method
1	AIF1L,CRABP1,IDI1,MEAK7,PLCD4	Random forest
2	AIF1L,CRABP1,IDI1,MEAK7,RASSF8	lightGBM
3	AIF1L,CRABP1,IDI1,MEAK7,RASSF8	Random forest
4	AIF1L,CRABP1,IDI1,MEAK7,RASSF8	KNN
5	IDI1,MEAK7,RASSF8,SMOC2	KNN
6	CRABP1,IDI1,MEAK7,RASSF8	lightGBM
7	CRABP1,IDI1,MEAK7,RASSF8	Random forest
8	IDI1,MEAK7,RASSF8,SMOC2	Random forest
9	IDI1,MEAK7,RASSF8,SMOC2	lightGBM
10	CRABP1,IDI1,MEAK7	Random forest

N

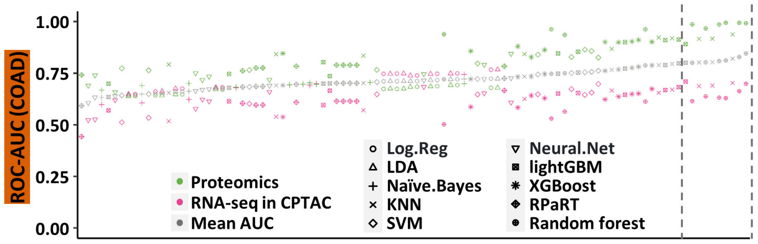

Top 10 Feature sets

Order	Feature set	MS method
1	CMBL,FMOD,ISLR	Random forest
2	CMBL,ISLR,NTM	Random forest
3	AEBP1,BGN,CMBL,ISLR	KNN
4	AEBP1,BGN,CMBL,ISLR	Random forest
5	CMBL,ISLR	Random forest
6	CMBL,FMOD,ISLR	KNN
7	AEBP1,CMBL	Random forest
8	AEBP1,CMBL,ISLR	KNN
9	AEBP1,CMBL,ISLR	Random forest
10	CMBL,FMOD,ISLR	lightGBM

O

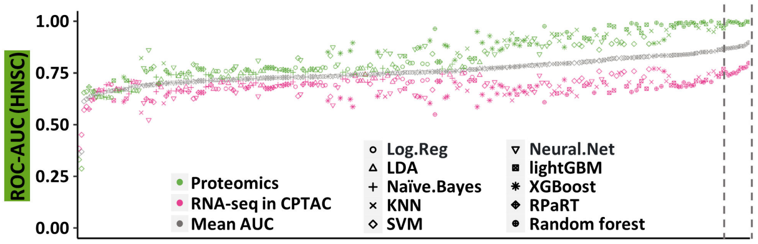

Top 10 Feature sets

Order	Feature set	MS method
1	LAMB3,P3H4,PLA2G2A,PRKCB,TBC1D10C	Random forest
2	CD79A,GIMAP5,LAMB3,PLA2G2A,TBC1D10C	Random forest
3	LAMB3,PLA2G2A,TBC1D10C	Random forest
4	LAMB3,P3H4,PLA2G2A,PRKCB,TBC1D10C	lightGBM
5	P3H4,PLA2G2A,TBC1D10C	Random forest
6	P3H4,PLA2G2A,TBC1D10C,ZBP1	Random forest
7	GIMAP5,JCHAIN,LAMB3,PLA2G2A	Random forest
8	GIMAP5,JCHAIN,LAMB3,PLA2G2A,TBC1D10C	Random forest
9	CTSG,P3H4,PLA2G2A,TBC1D10C,ZBP1	Random forest
10	LAMB3,TBC1D10C	Random forest

P

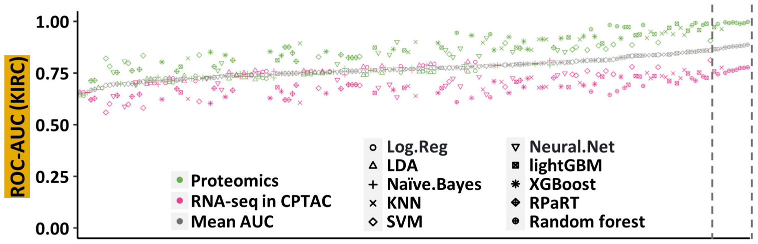

Top 10 Feature sets

Order	Feature set	MS method
1	CMA1,GCLC,GSTT2B,IL4I1,LGALS1	Random forest
2	CDCP1,FABP6,NFKBIE,SLC16A3	Random forest
3	CDCP1,NFKBIE,SLC16A3	Random forest
4	CMA1,GCLC,GSTT2B,IL4I1	Random forest
5	CDCP1,FABP6,GCLC,LGALS1,NFKBIE	Random forest
6	CDCP1,FABP6,GCLC,NFKBIE,SLC16A3	Random forest
7	CMA1,GCLC,GSTT2B,LGALS1	Random forest
8	CMA1,GCLC,GSTT2B,IL4I1,LGALS1	KNN
9	CMA1,GCLC,IL4I1	Random forest
10	CDCP1,FABP6,GCLC,NFKBIE,SLC16A3	lightGBM

Q

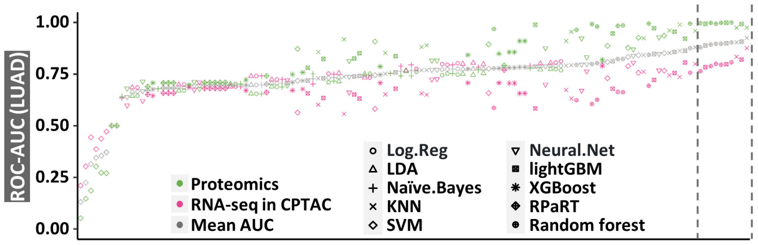

Top 10 Feature sets

Order	Feature set	MS method
1	AARS1,CPT1A,LAD1,STBD1	KNN
2	AARS1,CPT1A,LAD1,STBD1	lightGBM
3	AARS1,CPE,CPT1A,LAD1,STBD1	KNN
4	AARS1,CPT1A,LAD1,STBD1	Random forest
5	AARS1,CPE,CPT1A,LAD1,STBD1	lightGBM
6	CPT1A,LAD1	Random forest
7	CPT1A,LAD1,STBD1	Random forest
8	AARS1,CPE,CPT1A,LAD1,STBD1	Random forest
9	AARS1,CPE,CPT1A,STBD1	lightGBM
10	AARS1,CPE,CPT1A,STBD1	Random forest

R

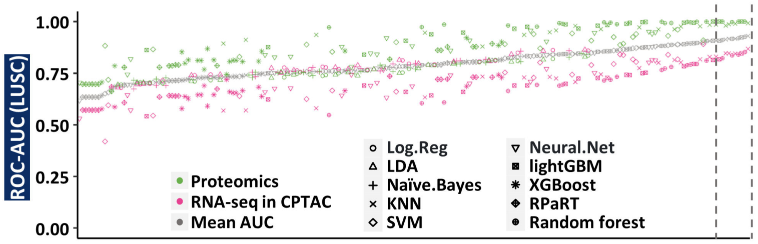

Top 10 Feature sets

Order	Feature set	MS method
1	ANGPTL4,MYO5C,PPL,RAB38,S100A2	KNN
2	ANGPTL4,MYO5C,PPL,RAB38,S100A2	Random forest
3	ANGPTL4,MYO5C,PPL,RAB38,S100A2	lightGBM
4	ANGPTL4,ANXA3,ASL,MYO5C,PPL	Random forest
5	ANGPTL4,ANXA3,ASL,MYO5C,PPL	KNN
6	ATP6V0D2,PPL,RAB38,S100A2,SCEL	KNN
7	ANXA1,ATP6V0D2,RAB38,S100A2,SCEL	Random forest
8	PPL,RAB38,S100A2,SCEL	KNN
9	ATP6V0D2,PPL,RAB38,S100A2,SCEL	Random forest
10	ANGPTL4,ANXA3,ASL,MYO5C,PPL	lightGBM

S

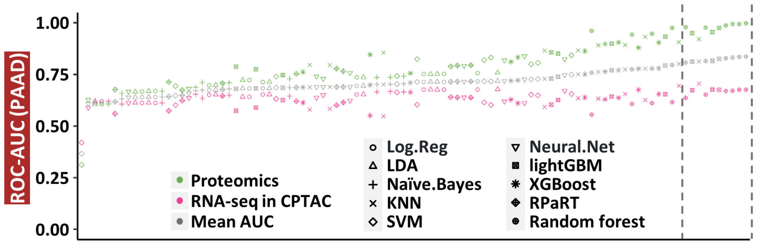

Top 10 Feature sets

Order	Feature set	MS method
1	CEACAM5,LAD1,SULT1C2,TPPP3	Random forest
2	CEACAM5,SULT1C2,TPPP3	Random forest
3	CEACAM5,LAD1,PRR15,TPPP3	Random forest
4	CEACAM5,LAD1,TPPP3	Random forest
5	CEACAM5,LAD1,SULT1C2,TPPP3	lightGBM
6	CEACAM5,LAD1,PRR15,TPPP3	lightGBM
7	LAD1,TPPP3	Random forest
8	CEACAM5,LAD1,SULT1C2,TPPP3	KNN
9	CEACAM5,SULT1C2,TPPP3	lightGBM
10	CEACAM5,TPPP3	Random forest

T

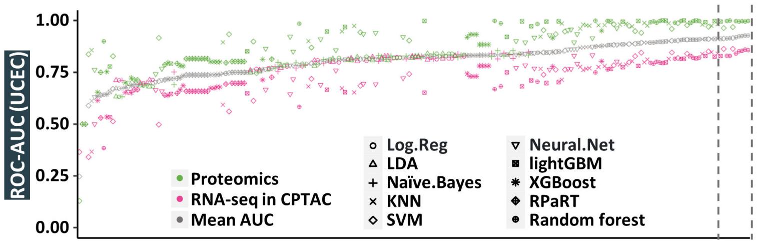

Top 10 Feature sets

Order	Feature set	MS method
1	CXCL5,DAPP1,EPHB2,HLA-DRA,MUC5B	Random forest
2	DAPP1,EPHB2,MUC5B	Random forest
3	DAPP1,EPHB2,HLA-DRA,MUC5B	Random forest
4	DAPP1,EPHB2,HLA-DRB1,MUC5B	Random forest
5	DAPP1,EPHB2,HLA-DMA,MUC5B	Random forest
6	DAPP1,EPHB2,HLA-DMA,HLA-DRA,MUC5B	SVM
7	CXCL5,DAPP1,EPHB2,MUC5B	Random forest
8	DAPP1,EPHB2,HLA-DMA,HLA-DRA,MUC5B	Random forest
9	DAPP1,EPHB2,HLA-DMA,MUC5B	SVM
10	DAPP1,EPHB2,HLA-DRA,MUC5B	lightGBM

Figure S5 cont'd

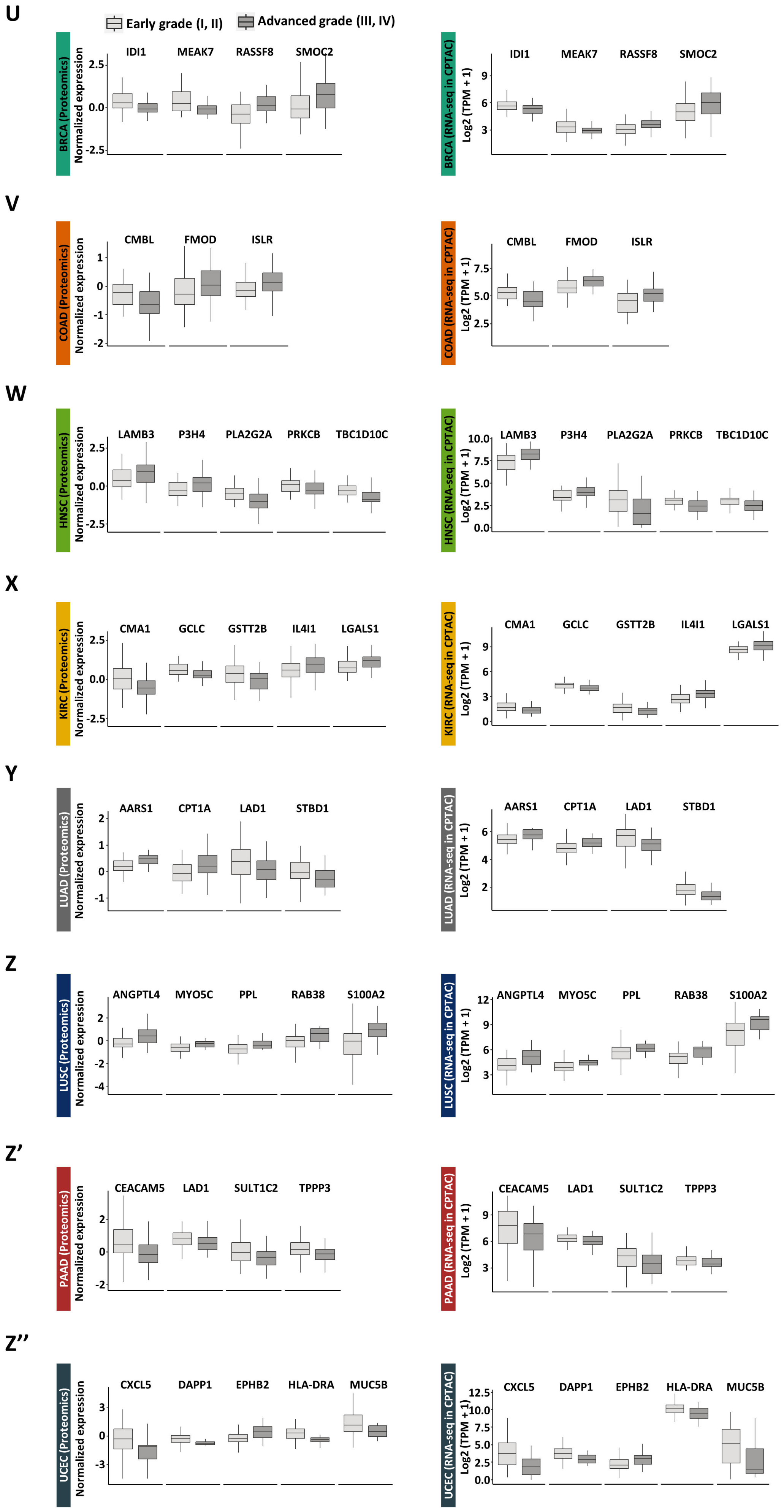

Figure S5 cont'd

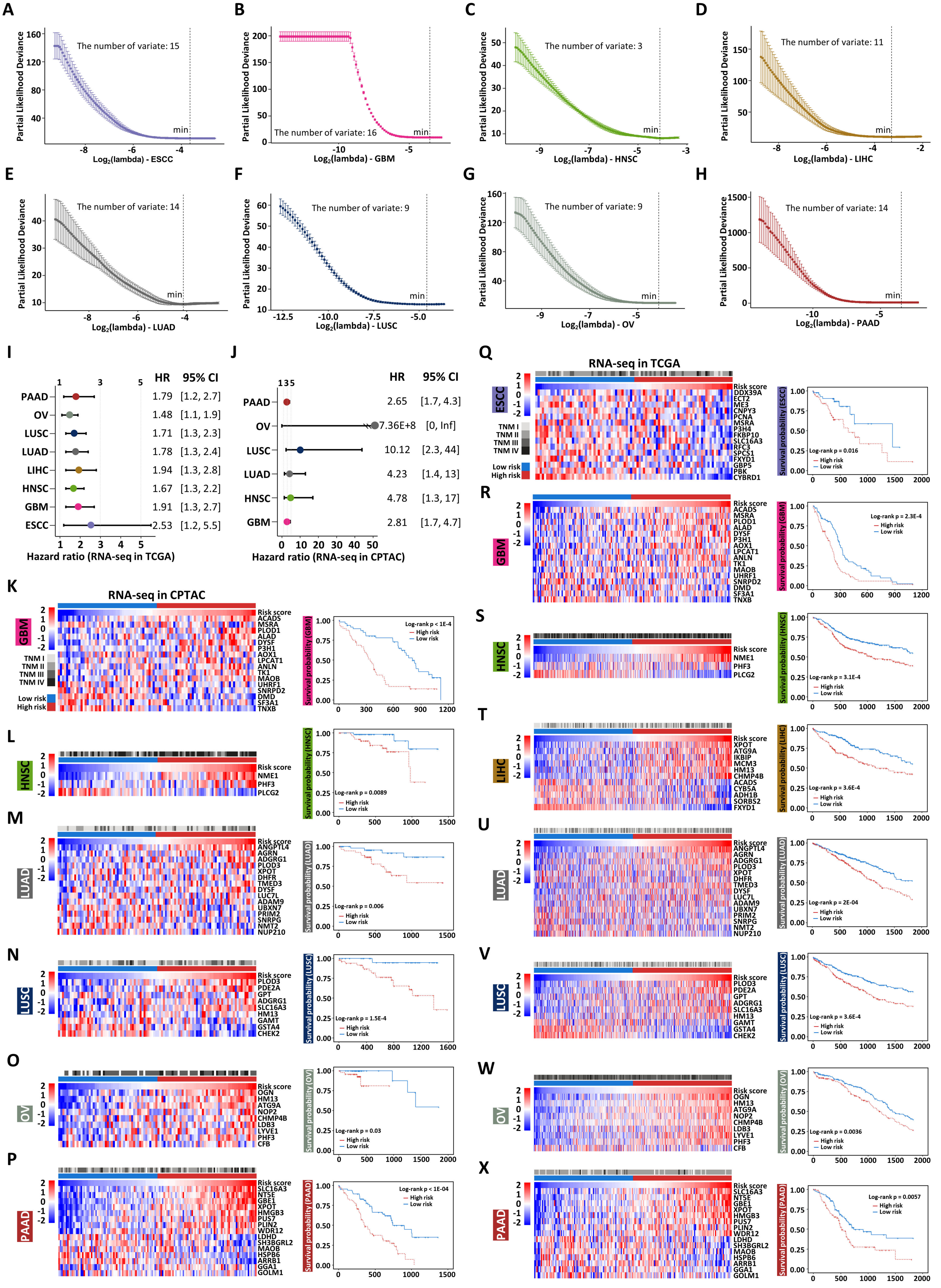
A
The number of variate: 15
Log2(lambda) - ESCC
B
The number of variate: 16
Log2(lambda) - GBM
C
The number of variate: 3
Log2(lambda) - HNSC
D
The number of variate: 11
Log2(lambda) - LIHC
E
The number of variate: 14
Log2(lambda) - LUAD
F
The number of variate: 9
Log2(lambda) - LUSC
G
The number of variate: 9
Log2(lambda) - OV
H
The number of variate: 14
Log2(lambda) - PAAD
Partial Likelihood Deviance
min
I
HR 95% CI
PAAD 1.79 [1.2, 2.7]
OV 1.48 [1.1, 1.9]
LUSC 1.71 [1.3, 2.3]
LUAD 1.78 [1.3, 2.4]
LIHC 1.94 [1.3, 2.8]
HNSC 1.67 [1.3, 2.2]
GBM 1.91 [1.3, 2.7]
ESCC 2.53 [1.2, 5.5]
Hazard ratio (RNA-seq in TCGA)
J
HR 95% CI
PAAD 2.65 [1.7, 4.3]
OV 7.36E+8 [0, Inf]
LUSC 10.12 [2.3, 44]
LUAD 4.23 [1.4, 13]
HNSC 4.78 [1.3, 17]
GBM 2.81 [1.7, 4.7]
Hazard ratio (RNA-seq in CPTAC)
K
RNA-seq in CPTAC
GBM
TNM I
TNM II
TNM III
TNM IV
Low risk
High risk
Risk score
ACADS MSRA PLOD1 ALAD DYSF P3H1 AOX1 LPCAT1 ANLN TK1 MAOB UHRF1 SNRPD2 DMD SF3A1 TNXB
Log-rank p < 1E-4
High risk
Low risk
Survival probability (GBM)
L
HNSC
Risk score
NME1 PHF3 PLCG2
Log-rank p = 0.0089
Survival probability (HNSC)
M
LUAD
Risk score
ANGPTL4 AGRN ADGRG1 PLOD3 XPOT DHFR TMED3 DYSF LUC7L ADAM9 UBXN7 PRIM2 SNRPG NMT2 NUP210
Log-rank p = 0.006
Survival probability (LUAD)
N
LUSC
Risk score
PLOD3 PDE2A GPT ADGRG1 SLC16A3 HM13 GAMT GSTA4 CHEK2
Log-rank p = 1.5E-4
Survival probability (LUSC)
O
OV
Risk score
OGN HM13 ATG9A NOP2 CHMP4B LDB3 LYVE1 PHF3 CFB
Log-rank p = 0.03
Survival probability (OV)
P
PAAD
Risk score
SLC16A3 NT5E GBE1 XPOT HMGB3 PUS7 PLIN2 WDR12 LDHD SH3BGRL2 MAOB HSPB6 ARRB1 GGA1 GOLM1
Log-rank p < 1E-04
Survival probability (PAAD)
Q
RNA-seq in TCGA
ESCC
TNM I
TNM II
TNM III
TNM IV
Low risk
High risk
Risk score
DDX39A ECT2 ME3 CNPY3 PCNA MSRA P3H4 FKBP10 SLC16A3 RFC3 SPCS1 FXYD1 GBP5 PBK CYBRD1
Log-rank p = 0.016
Survival probability (ESCC)
R
GBM
Risk score
ACADS MSRA PLOD1 ALAD DYSF P3H1 AOX1 LPCAT1 ANLN TK1 MAOB UHRF1 SNRPD2 DMD SF3A1 TNXB
Log-rank p = 2.3E-4
Survival probability (GBM)
S
HNSC
Risk score
NME1 PHF3 PLCG2
Log-rank p = 3.1E-4
Survival probability (HNSC)
T
LIHC
Risk score
XPOT ATG9A IKBIP MCM3 HM13 CHMP4B ACADS CYB5A ADH1B SORBS2 FXYD1
Log-rank p = 3.6E-4
Survival probability (LIHC)
U
LUAD
Risk score
ANGPTL4 AGRN ADGRG1 PLOD3 XPOT DHFR TMED3 DYSF LUC7L ADAM9 UBXN7 PRIM2 SNRPG NMT2 NUP210
Log-rank p = 2E-04
Survival probability (LUAD)
V
LUSC
Risk score
PLOD3 PDE2A GPT ADGRG1 SLC16A3 HM13 GAMT GSTA4 CHEK2
Log-rank p = 3.6E-4
Survival probability (LUSC)
W
OV
Risk score
OGN HM13 ATG9A NOP2 CHMP4B LDB3 LYVE1 PHF3 CFB
Log-rank p = 0.0036
Survival probability (OV)
X
PAAD
Risk score
SLC16A3 NT5E GBE1 XPOT HMGB3 PUS7 PLIN2 WDR12 LDHD SH3BGRL2 MAOB HSPB6 ARRB1 GGA1 GOLM1
Log-rank p = 0.0057
Survival probability (PAAD)

Figure S6

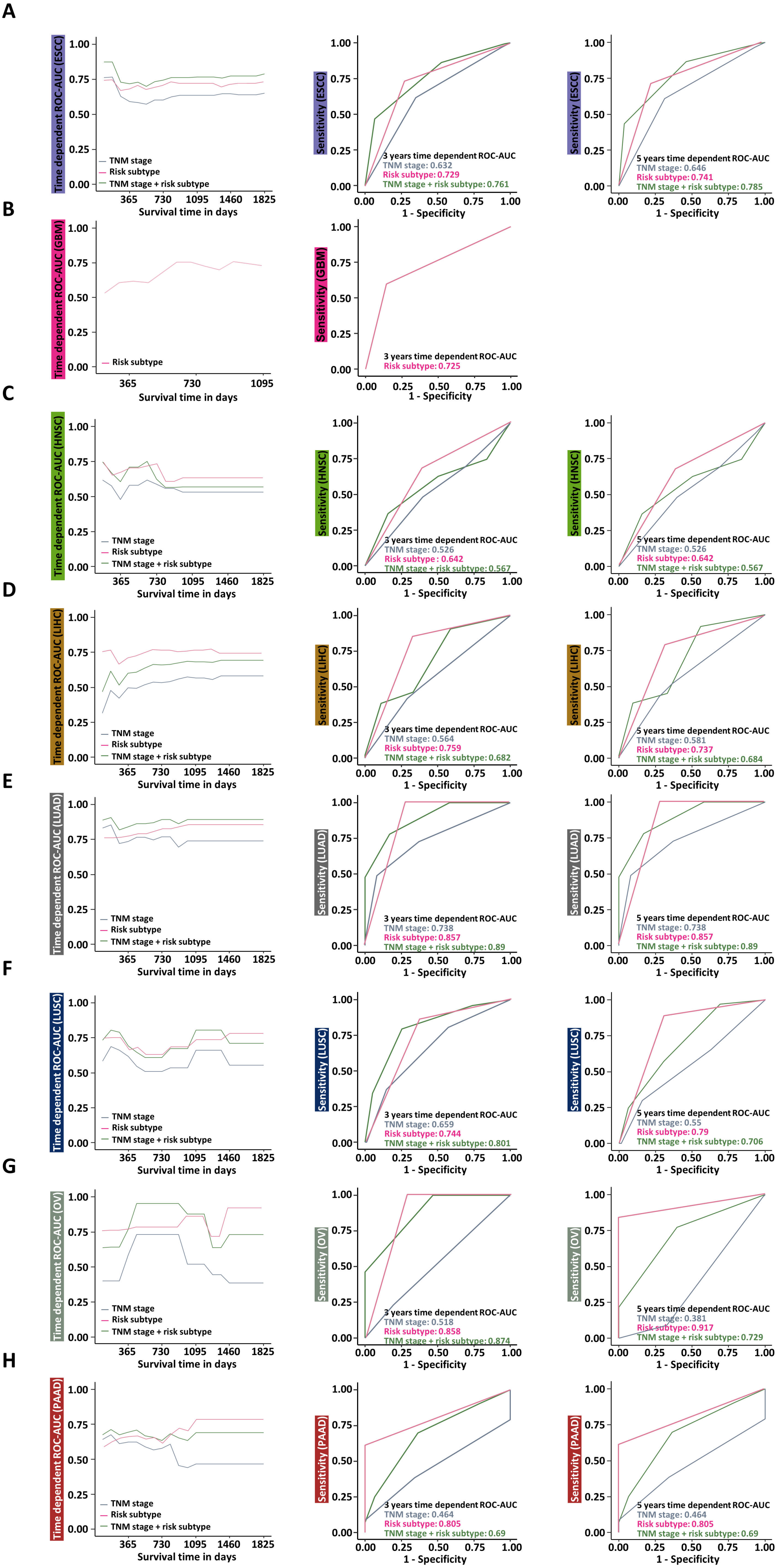
A
Time dependent ROC-AUC (ESCC)
TNM stage
Risk subtype
TNM stage + risk subtype
Survival time in days
Sensitivity (ESCC)
1 - Specificity
3 years time dependent ROC-AUC
TNM stage: 0.632
Risk subtype: 0.729
TNM stage + risk subtype: 0.761
5 years time dependent ROC-AUC
TNM stage: 0.646
Risk subtype: 0.741
TNM stage + risk subtype: 0.785
B
Time dependent ROC-AUC (GBM)
Risk subtype
Sensitivity (GBM)
3 years time dependent ROC-AUC
Risk subtype: 0.725
C
Time dependent ROC-AUC (HNSC)
Sensitivity (HNSC)
3 years time dependent ROC-AUC
TNM stage: 0.526
Risk subtype : 0.642
TNM stage + risk subtype: 0.567
5 years time dependent ROC-AUC
TNM stage: 0.526
Risk subtype: 0.642
TNM stage + risk subtype: 0.567
D
Time dependent ROC-AUC (LIHC)
Sensitivity (LIHC)
3 years time dependent ROC-AUC
TNM stage: 0.564
Risk subtype: 0.759
TNM stage + risk subtype: 0.682
5 years time dependent ROC-AUC
TNM stage: 0.581
Risk subtype: 0.737
TNM stage + risk subtype: 0.684
E
Time dependent ROC-AUC (LUAD)
Sensitivity (LUAD)
3 years time dependent ROC-AUC
TNM stage: 0.738
Risk subtype: 0.857
TNM stage + risk subtype: 0.89
5 years time dependent ROC-AUC
TNM stage: 0.738
Risk subtype: 0.857
TNM stage + risk subtype: 0.89
F
Time dependent ROC-AUC (LUSC)
Sensitivity (LUSC)
3 years time dependent ROC-AUC
TNM stage: 0.659
Risk subtype: 0.744
TNM stage + risk subtype: 0.801
5 years time dependent ROC-AUC
TNM stage: 0.55
Risk subtype: 0.79
TNM stage + risk subtype: 0.706
G
Time dependent ROC-AUC (OV)
Sensitivity (OV)
3 years time dependent ROC-AUC
TNM stage: 0.518
Risk subtype: 0.858
TNM stage + risk subtype: 0.874
5 years time dependent ROC-AUC
TNM stage: 0.381
Risk subtype: 0.917
TNM stage + risk subtype: 0.729
H
Time dependent ROC-AUC (PAAD)
Sensitivity (PAAD)
3 years time dependent ROC-AUC
TNM stage: 0.464
Risk subtype: 0.805
TNM stage + risk subtype: 0.69
5 years time dependent ROC-AUC
TNM stage: 0.464
Risk subtype: 0.805
TNM stage + risk subtype: 0.69

Figure S7

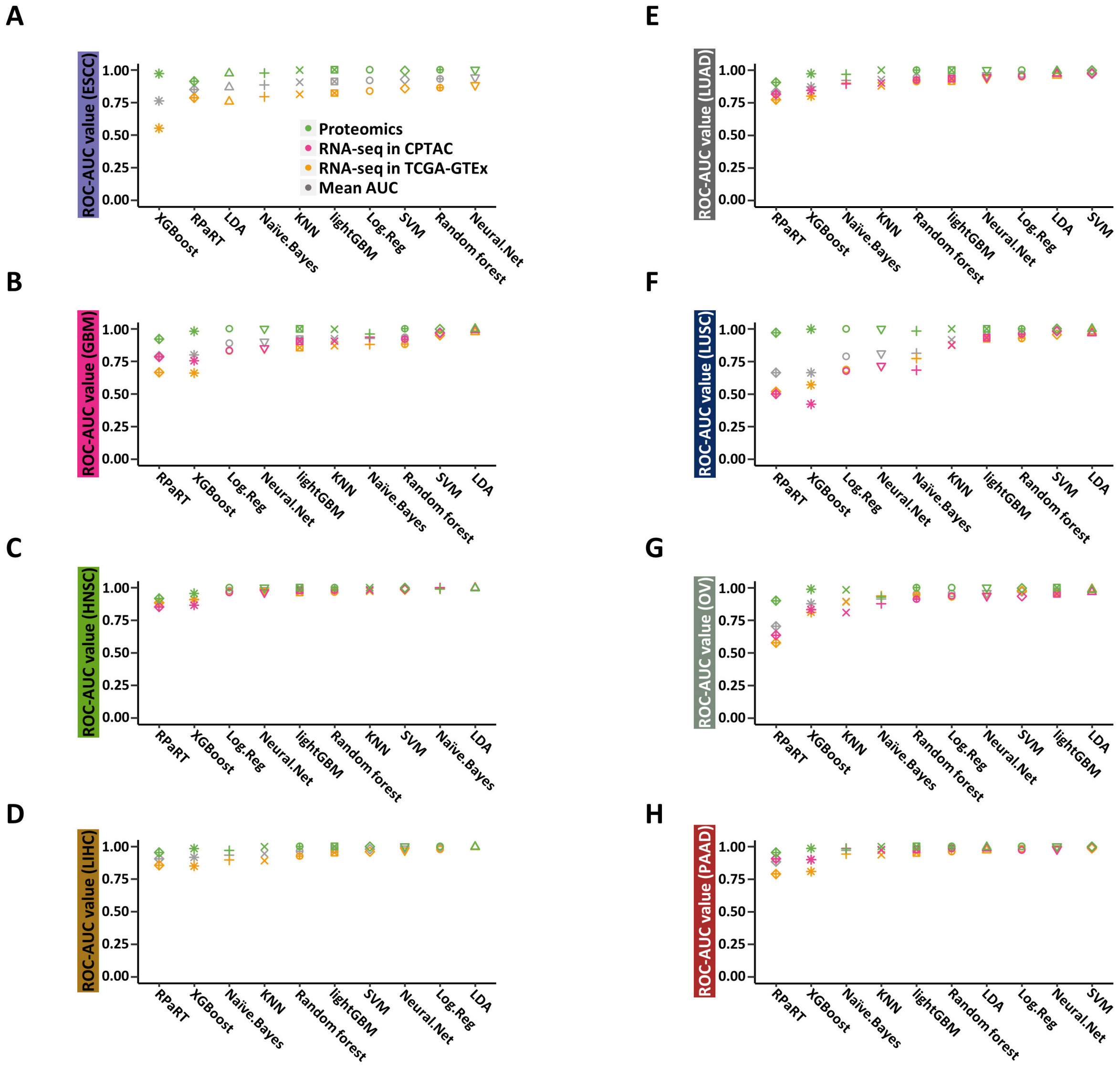
A
ROC-AUC value (ESCC)
1.00
0.75
0.50
0.25
0.00
Proteomics
RNA-seq in CPTAC
RNA-seq in TCGA-GTEx
Mean AUC
XGBoost RPaRT LDA Naïve.Bayes KNN lightGBM Log.Reg SVM Random forest Neural.Net
B
ROC-AUC value (GBM)
RPaRT XGBoost Log.Reg Neural.Net lightGBM KNN Naïve.Bayes Random forest SVM LDA
C
ROC-AUC value (HNSC)
RPaRT XGBoost Log.Reg Neural.Net lightGBM Random forest KNN SVM Naïve.Bayes LDA
D
ROC-AUC value (LIHC)
RPaRT XGBoost Naïve.Bayes KNN Random forest lightGBM SVM Neural.Net Log.Reg LDA
E
ROC-AUC value (LUAD)
RPaRT XGBoost Naïve.Bayes KNN Random forest lightGBM Neural.Net Log.Reg LDA SVM
F
ROC-AUC value (LUSC)
RPaRT XGBoost Log.Reg Neural.Net Naïve.Bayes KNN lightGBM Random forest SVM LDA
G
ROC-AUC value (OV)
RPaRT XGBoost KNN Naïve.Bayes Random forest Log.Reg Neural.Net SVM lightGBM LDA
H
ROC-AUC value (PAAD)
RPaRT XGBoost Naïve.Bayes KNN lightGBM Random forest LDA Log.Reg Neural.Net SVM

Figure S8

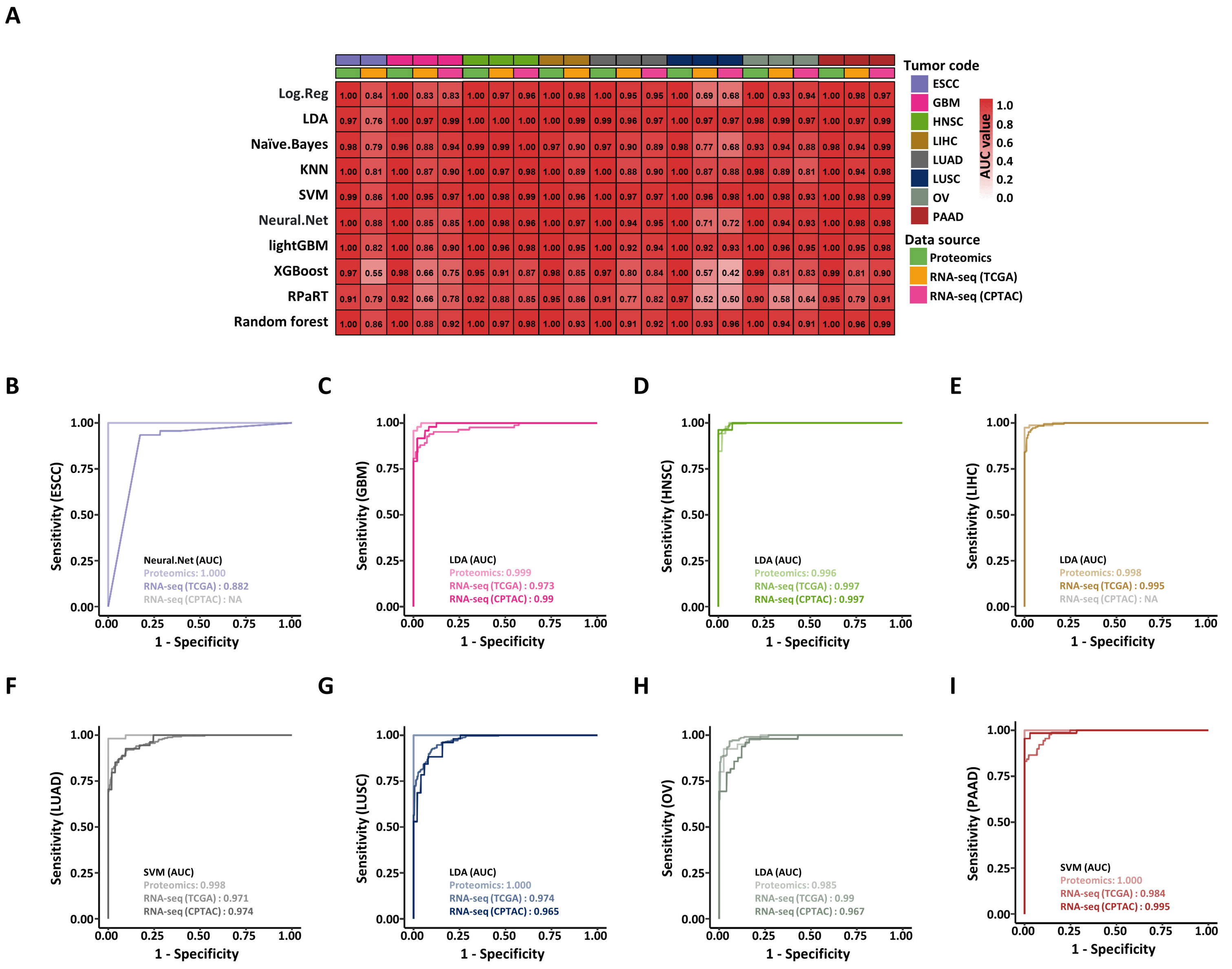
A
Tumor code
ESCC
GBM
HNSC
LIHC
LUAD
LUSC
OV
PAAD
AUC value
1.0
0.8
0.6
0.4
0.2
0.0
Data source
Proteomics
RNA-seq (TCGA)
RNA-seq (CPTAC)
Log.Reg 1.00 0.84 1.00 0.83 0.83 1.00 0.97 0.96 1.00 0.98 1.00 0.95 0.95 1.00 0.69 0.68 1.00 0.93 0.94 1.00 0.98 0.97
LDA 0.97 0.76 1.00 0.97 0.99 1.00 1.00 1.00 1.00 0.99 0.99 0.96 0.97 1.00 0.97 0.97 0.98 0.99 0.97 1.00 0.97 0.99
Naïve.Bayes 0.98 0.79 0.96 0.88 0.94 0.99 0.99 1.00 0.97 0.90 0.97 0.90 0.89 0.98 0.77 0.68 0.93 0.94 0.88 0.98 0.94 0.99
KNN 1.00 0.81 1.00 0.87 0.90 1.00 0.97 0.98 1.00 0.89 1.00 0.88 0.90 1.00 0.87 0.88 0.98 0.89 0.81 1.00 0.94 0.98
SVM 0.99 0.86 1.00 0.95 0.97 1.00 0.98 0.99 1.00 0.96 1.00 0.97 0.97 1.00 0.96 0.98 1.00 0.98 0.93 1.00 0.98 0.99
Neural.Net 1.00 0.88 1.00 0.85 0.85 1.00 0.98 0.96 1.00 0.97 1.00 0.94 0.95 1.00 0.71 0.72 1.00 0.94 0.93 1.00 0.98 0.98
lightGBM 1.00 0.82 1.00 0.86 0.90 1.00 0.96 0.98 1.00 0.95 1.00 0.92 0.94 1.00 0.92 0.93 1.00 0.96 0.95 1.00 0.95 0.98
XGBoost 0.97 0.55 0.98 0.66 0.75 0.95 0.91 0.87 0.98 0.85 0.97 0.80 0.84 1.00 0.57 0.42 0.99 0.81 0.83 0.99 0.81 0.90
RPaRT 0.91 0.79 0.92 0.66 0.78 0.92 0.88 0.85 0.95 0.86 0.91 0.77 0.82 0.97 0.52 0.50 0.90 0.58 0.64 0.95 0.79 0.91
Random forest 1.00 0.86 1.00 0.88 0.92 1.00 0.97 0.98 1.00 0.93 1.00 0.91 0.92 1.00 0.93 0.96 1.00 0.94 0.91 1.00 0.96 0.99
B
Sensitivity (ESCC)
1 - Specificity
Neural.Net (AUC)
Proteomics: 1.000
RNA-seq (TCGA) : 0.882
RNA-seq (CPTAC) : NA
C
Sensitivity (GBM)
1 - Specificity
LDA (AUC)
Proteomics: 0.999
RNA-seq (TCGA) : 0.973
RNA-seq (CPTAC) : 0.99
D
Sensitivity (HNSC)
1 - Specificity
LDA (AUC)
Proteomics: 0.996
RNA-seq (TCGA) : 0.997
RNA-seq (CPTAC) : 0.997
E
Sensitivity (LIHC)
1 - Specificity
LDA (AUC)
Proteomics: 0.998
RNA-seq (TCGA) : 0.995
RNA-seq (CPTAC) : NA
F
Sensitivity (LUAD)
1 - Specificity
SVM (AUC)
Proteomics: 0.998
RNA-seq (TCGA) : 0.971
RNA-seq (CPTAC) : 0.974
G
Sensitivity (LUSC)
1 - Specificity
LDA (AUC)
Proteomics: 1.000
RNA-seq (TCGA) : 0.974
RNA-seq (CPTAC) : 0.965
H
Sensitivity (OV)
1 - Specificity
LDA (AUC)
Proteomics: 0.985
RNA-seq (TCGA) : 0.99
RNA-seq (CPTAC) : 0.967
I
Sensitivity (PAAD)
1 - Specificity
SVM (AUC)
Proteomics: 1.000
RNA-seq (TCGA) : 0.984
RNA-seq (CPTAC) : 0.995

Figure S9
