# Supplementary material for: Integrated Analysis of Proteome and Transcriptome Profiling Reveals Pan-Cancer-Associated Pathways and Molecular Biomarkers
Source: Mol Cell Proteomics. 2025 Jan 28;24(3):100919. doi: 10.1016/j.mcpro.2025.100919 (PMC11907456; doi:10.1016/j.mcpro.2025.100919)
Supplement: Supplementary Figure Legends [file mmc7.docx]

**Supplementary figure legends**

**Supplementary figure 1. Compendium of proteomic and transcriptomic datasets**

**(A)** Proteins detected in proteomic data as described in Fig. 1A at different cutoff.

**(B)** Gene ontology analysis for dataset Prot5 as shown in (A). Yellow, green, and blue bars represent enriched terms of biological process (BP), molecular function (MF), and cellular component (CC), respectively.

**(C)** Protein-protein interaction enrichment analysis for unique proteins in GBM tissues.

**(D)** The distribution of spearman's correlation is shown by Pie chart. Darker shades represent spearman’s correlation with *P* value < 0.01.

**(E)** Annotated gene sets (Gene Ontology) across 9,057 genes ranked by Spearman gene-wise correlation between protein and mRNA. Top 5 most significant gene sets are shown in GSEA analysis results.

**(F)** Histogram of gene-wise Spearman correlations of mRNA and protein expression in each cancer type.

**Supplementary figure 2. Dysregulated genes and pathways were identified by transcriptomic and proteomic analysis**

**(A)** Tissue-specific mRNAs as detected in RNA-seq sourced from CPTAC dataset are shown by boxplot. Within each color group, lighter shades represent normal or ANTs, while darker shades represent tumor tissues. *P* value was calculated by two-sided wilcoxon rank-sum test. The middle bar represents the median, and the box represents the interquartile range. Bars extend to 1.5× the interquartile range (****P* < 0.001).

**(B)** The expression of tissue-specific mRNAs and proteins in GBM (ELAVL3, GABBR2), KIRC (NPHS2, SLC22A13), LUAD/LUSC (SFTPA1, SCGB1A1), PAAD (AMY2A, CEL), and UCEC (PGR, SFRP4) are shown by boxplot.

**(C)** The overall protein abundance is shown by boxplot as described in Fig. 2A.

**(D)** Differential expression analysis showing up- and down-regulated mRNAs in RNA-seq sourced from TCGA-GTEx dataset across all 13 cancer types. Red and blue colors represent mRNAs with *Q* value (Benjamini-Hochberg (BH) adjusted *P* value) < 0.01 and fold change (FC) > 1.5. The value represents the number of differentially expressed mRNAs. *P* value was calculated by two-sided wilcoxon rank-sum test.

**(E)** Protein abundance of UGs and DGs is shown by heatmap.

**(F)** The significance of enrichment for gene sets from Hallmark, KEGG, and wikiPathway with UGs and DGs in each cancer type is shown by heatmap. The *Q* value of the gene set must be less than 0.001, and the number of intersections with UGs and DGs must be at least 15. The enrichment results of UGs and DGs are represented by red and blue color, respectively.

**(G-L)** Diagram representing processing of intron-containing pre-mRNA (G), type I interferons signaling (H), type II interferons signaling (I), fatty acid oxidation (J), complement and coagulation cascades (K), and complement activation (L), with the degree of differential expression of differential proteins and mRNAs in corresponding cancer types. Red and blue colors represent significantly over-presented and under-presented genes in cancer, respectively. The top and bottom square represent mRNAs and protein, respectively.

**(M)** Protein-protein interaction networks and function enrichment analysis of PCUGs and PCDGs. Red and blue circles represent PCUGs and PCDGs, respectively.

**Supplementary Figure 3. RRM2 and ADH1B were identified as potential pan-cancer diagnostic markers**

**(A, B)** The abundance of RRM2 (A) and ADH1B (B) before and after standardization in proteomics data in CPTAC, RNA-seq in TCGA-GTEx, and RNA-seq in CPTAC. Red and blue colors represent cancer and non-cancer (normal or ANTs) samples, respectively.

**(C)** ROC-AUCs calculated by 10 machine learning algorithms (Log.Reg, LDA, Naïve.Bayes, KNN, SVM, Neural.Net, lightGBM, XGBoost, RPaRT, and Random forest) with 100 rounds of five-fold cross-validation (CV) based on RRM2 and ADH1B z-score value in transcriptomic data sourced from TCGA-GTEx is shown by boxplot (left panel). Final ROC-AUCs calculated by 10 machine learning algorithms are shown by table (right panel).

**(D)** ROC curve (left panel) and PR curve (right panel) of the Neural.Net model constructed by transcriptomic z-score value (TCGA-GTEx) of RRM2 and ADH1B in all cancer types. Yellow, green, and red represent RNA-seq in TCGA-GTEx (training cohort), proteomic data (testing cohort), and RNA-seq in CPTAC (testing cohort), respectively.

**(E, G, I)** Heatmap of CPTAC proteomic (E), TCGA-GTEx transcriptomic (G), and CPTAC transcriptomic (I) PR-AUCs of the prediction model constructed by 10 machine learning algorithms with 100 rounds of three-fold CV based on proteomic z-score value of RRM2 and ADH1B in each cancer type.

**(F, H, J)** CPTAC proteomic (F), TCGA-GTEx transcriptomic (H) and CPTAC transcriptomic (J) PR curve of the prediction model constructed by best learning algorithms in (F).

**(K, M, O)** Heatmap of TCGA-GTEx transcriptomic (K), CPTAC proteomic (M), and CPTAC transcriptomic (O) ROC-AUCs of the prediction model constructed by 10 machine learning algorithms with 100 rounds of three-fold cross-validation (CV) based on TCGA-GTEx transcriptomic z-score value of RRM2 and ADH1B in each cancer type.

**(L, N, P)** Proteomic CPTAC (L), TCGA-GTEx transcriptomic (N) and CPTAC transcriptomic (P) ROC curve of the prediction model constructed by best learning algorithms in (L).

**(Q, S, U)** Heatmap of TCGA-GTEx transcriptomic (Q), CPTAC proteomic (S), and CPTAC transcriptomic (U) PR-AUCs of the prediction model constructed by 10 machine learning algorithms with 100 rounds of three-fold cross-validation (CV) based on TCGA-GTEx transcriptomic z-score value of RRM2 and ADH1B in each cancer type.

**(R, T, V)** Proteomic CPTAC (R), TCGA-GTEx transcriptomic (T) and CPTAC transcriptomic (V) PR curve of the prediction model constructed by best learning algorithms in (R).

**(W-Z)** Kaplan-Meier survival analysis of overall survival (OS) between the high and low expression RRM2 (W, X) or ADH1B (Y, Z) groups in the proteomic data sourced from CPTAC (W, Y) and transcriptomic data sourced from TCGA-GTEx (X, Z). *P* value was calculated by two-sided log-rank test.

**Supplementary Figure 4. Dysregulated genes and pathways were identified during cancer progression**

**(A)** The distribution of the number of samples in each TNM stage in each cancer type. The four different shades of gray represent TNM stages I, II, III, and IV, respectively.

**(B)** The number of differential expressed proteins across 8 cancer types as indicated are shown by pie chart. *P* value was calculated by one-way analysis of variance.

**(C)** Expression trend analysis of differentially expressed proteins during cancer progression (TNM stage) in each cancer is shown by heatmap.

**(D)** Correlation of signature score between complement and coagulation pathway and epithelial mesenchymal transformation process in COAD, LUSC, and PAAD. The correlation coefficient was calculated by Pearson algorithm.

**(E, F)** Boxplot of the abundance of proteins contained in enrichment results of the complement and coagulation pathway (E) and EMT process (F) in trend clusters of COAD.

**(G-M)**. The abundance of proteins contained in enrichment results of complement and coagulation pathway in LUSC trend clusters (G), epithelial mesenchymal transition in LUSC trend clusters (H), complement and coagulation pathway in PAAD trend clusters (I), epithelial mesenchymal transition in PAAD trend clusters (J), fatty acids and lipoproteins transport in LUAD trend clusters (K), oxidative phosphorylation in HNSC trend clusters (L), and ciliary landscape in UCEC trend clusters (M) is shown by heatmap.

**(N, O)** The number of proteins with upward (N) and downward (O) trends according to cancer progression in each trend cluster of each cancer is shown in table.

**(P, Q)** UpSet plot of the intersections of continuously up- (P) and down-regulated (Q) proteins in different tumor stage.

**(R, S)** KEGG pathways (R) and hallmark get sets (S) enriched in PCCUPs and PCCDPs. Red and blue dots indicate pathways enriched in PCCUPs and PCCDPs, respectively.

**Supplementary Figure 5. Establishment and validation of tumor stage classification models**

**(A, B)** The distribution of the number of samples in early and advanced stages of each tumor type in proteomic (A) and transcriptomic (B) data sourced from CPTAC. TNM I and II stages were classified as early stages, while TNM III and IV stages as advanced stages. The light and dark gray represent early and advanced stage, respectively.

**(C)** The number of differentially expressed proteins and mRNAs across 8 cancer types as indicated are shown by bar plots. Within each color group, lighter shades represent differentially expressed mRNAs, while darker shades represent differentially expressed proteins. *P* value was calculated by two-sided Wilcoxon rank-sum test.

**(D)** The intersection of the differentially expressed mRNAs and proteins as shown in (C) is shown by Venn diagram.

**(E-L)** The frequency of the candidate feature sets with 100 times feature selections in BRCA (E), COAD (F), HNSC (G), KIRC (H), LUAD (I), LUSC (J), PAAD (K), and UCEC (L). Green, purple, orange, yellow, and blue color indicates the maximum number of features is 1, 2, 3, 4, and 5, respectively.

**(M-T)**. ROC-AUCs calculated by 10 machine learning algorithms as indicated combined with the candidate feature sets in BRCA (M), COAD (N), HNSC (O), KIRC (P), LUAD (Q), LUSC (R), PAAD (S), and UCEC (T) are shown by dot plots (left panel). Top 10 stage classification models and corresponding feature sets are shown in table (right panel). Green and red colors represent ROC-AUCs for proteomic and transcriptomic data sourced from CPTAC, respectively, while gray color represents the average of ROC-AUCs for the above two datasets.

**(U-Z’’)**. The abundance of proteins (left panel) and mRNAs (right panel) involved in stage classification biomarker panels in BRCA (U), COAD (V), HNSC (W), KIRC (X), LUAD (Y), LUSC (Z), PAAD (Z’), and UCEC (Z’’) is shown by boxplot.

**Supplementary Figure 6. Establishment and validation of risk stratification model**

**(A-H)** Variable selection processes of LASSO-Cox regression model and the corresponding number of variables in ESCC (A), GBM (B), HNSC (C), LIHC (D), LUAD (E), LUSC (F), OV (G), and PAAD (H).

**(I, J)** The hazard ratios (HRs) of high-risk groups of all 8 cancer types in transcriptomic data sourced from TCGA-GTEx (I) and CPTAC (J) (left panel) are shown by forest plots, and the 95% confidence intervals (CIs) of corresponding HRs are shown by table (right panel).

**(K-P)** Heatmap (left panel) of the abundance of risk mRNAs (CPTAC) involved in risk prognostic risk stratification models and Kaplan-Meier plot (right panel) displaying the prognosis of high-risk group in GBM (K), HNSC (L), LUAD (M), LUSC (N), OV (O), and PAAD (P).

**(Q-X)** Heatmap (left panel) of the abundance of risk mRNAs (TCGA-GTEx) involved in risk prognostic risk stratification models and Kaplan-Meier plot (right panel) displaying the prognosis of high-risk group in ESCC (Q), GBM (R), HNSC (S), LIHC (T), LUAD (U), LUSC (V), OV (W), and PAAD (X).

**Supplementary Figure 7. Analysis of time-dependent ROC**

(**A-H**) Area under the time-dependent ROC curve (TDROC) for OS (left panel), TDROC for OS at three years (middle panel), and TDROC for OS at five years (right panel) in ESCC (A), GBM (B), HNSC (C), LIHC (D), LUAD (E), LUSC (F), OV (G) and PAAD (H). There was no TNM stage in GBM, and the patient's prognosis was less than 3 years, so some analysis results were missing. Green and purple color represent the TDROC of “Risk group + TNM” and TNM stage, respectively.

**Supplementary Figure 8. ROC-AUCs calculated by 10 machine learning algorithms**

**(A-H)**. ROC-AUCs calculated by 10 machine learning algorithms combined with risk gene sets in ESCC (A), GBM (B), HNSC (C), LIHC (D), LUAD (E), LUSC (F), OV (G), and PAAD (H) are shown by dot plots.

**Supplementary Figure 9. Construction of the best prognostic risk stratification model for each cancer type**

**(A)** ROC-AUC values calculated by the best prognostic risk stratification models for all cancer types are shown by heatmap. Green, yellow, and red colors represent ROC-AUCs for proteomic data sourced from CPTAC, transcriptomic data sourced from TCGA-GTEx, and transcriptomic data sourced from CPTAC, respectively.

**(B-I)**. ROC curves of the best prognostic risk stratification models in ESCC (B), GBM (C), HNSC (D), LIHC (E), LUAD (F), LUSC (G), OV (H), and PAAD (I). The three different shades represent proteomic data sourced from CPTAC, transcriptomic data sourced from TCGA-GTEx, and transcriptomic data sourced from CPTAC, respectively.
